# Hydrogen‐Bond‐Coupling Interfacial Microenvironment Enables Fast Charging of Sodium‐Ion Batteries Over a Wide Temperature Range

**DOI:** 10.1002/advs.202517001

**Published:** 2025-10-27

**Authors:** Jin‐Ling Liu, Xiao‐Tong Wang, Denglong Chen, Zhen‐Yi Gu, Yi‐Fei Liu, Yan Zhuang, Yong‐Li Heng, Hang Li, Xing‐Long Wu

**Affiliations:** ^1^ College of Environmental and Resource Sciences and College of Carbon Neutral Modern Industry Fujian Normal University Fuzhou 350007 P. R. China; ^2^ State Key Laboratory of Integrated Optoelectronics MOE Key Laboratory for UV Light‐Emitting Materials and Technology Northeast Normal University Changchun 130024 P. R. China; ^3^ Quangang Petrochemical Research Institute Fujian Normal University Quanzhou 362801 P. R. China

**Keywords:** cathode, hydrogen‐bond‐coupling, interface, sodium‐ion batteries, wide‐temperature

## Abstract

With the deepening global energy transition and expanding diversified application scenarios, developing sodium‐ion batteries with both fast‐charging capability and wide‐temperature adaptability has become urgent. However, the core challenge lies in constructing a stable cathode‐electrolyte interface (CEI). Traditional strategies overly rely on electrolyte formulation adjustments while neglecting intrinsic material surface engineering. This study innovatively proposes a hydrogen‐bond ‐coupling mechanism between surface hydroxyl groups on the cathode and electrolyte molecules, precisely tuning the interface microenvironment to synergistically resolve conflicts between high/low‐temperature interfacial failures. Specifically, hydrogen bonding induces preferential decomposition of fluoroethylene carbonate (FEC) to form a NaF‐rich CEI layer that suppresses parasitic reactions, and strengthened Na^⁺^‐interface interactions significantly reduce ion diffusion energy barriers. Validated on the Na_3_V_2_(PO_4_)_3_ cathode, this strategy endows exceptional performance across an ultra‐wide temperature range: achieving 80% charging only takes 38.8 s at 60C at room temperature, retaining 84.17% capacity after 1600 cycles at 80 °C and 10C, and operating normally even at an extremely low temperature of −80 °C. This work breaks through conventional interface optimization paradigms, providing a universal new strategy for interface chemical design of high‐performance electrode materials.

## Graphical abstract

The surface hydroxyl groups on Na_3_V_2_(PO_4_)_3_ enhance its adsorption energy with electrolyte molecules through hydrogen‐bond interactions. This unique coupling mechanism facilitates the formation of an exceptionally stable cathode‐electrolyte interface layer, endowing the Na_3_V_2_(PO_4_)_3_ cathode with remarkable fast‐charging capability across an ultra‐wide temperature range (−80–80 °C).

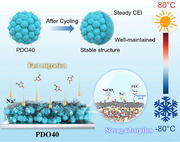

## 1 Introduction

High‐performance sodium‐ion batteries (SIBs) face two critical bottlenecks in their application for large‐scale energy storage and electric vehicles: fast charging capability and wide‐temperature adaptability.^[^ ^1^, ^2^ ^]^ Fast‐charging performance directly impacts user experience and grid response speed, while tolerance to a broad temperature range is essential for reliable operation under diverse global climates.^[^ ^3^, ^4^ ^]^ Conventional batteries struggle under extreme conditions: low temperatures cause a dramatic drop in ionic conductivity and a surge in interface impedance, leading to severe performance degradation or failure; high temperatures accelerate side reactions and structural decay at interfaces, severely limiting cycle life and safety.^[^ ^5^, ^6^ ^]^

The intertwined challenges of fast charging and wide‐temperature adaptability ultimately hinge on a fundamental electrochemical bottleneck: the dynamic evolution of the solid–liquid interface between the cathode material and electrolyte, namely the formation mechanism, evolution, and intrinsic physicochemical properties of the cathode‐electrolyte interface (CEI) layer.^[^ ^7^, ^8^ ^]^ The stability, ion transport kinetics, and electrochemical compatibility of this interface fundamentally determine the rate capability of SIBs, energy efficiency, cycling stability, and functional integrity under extreme conditions.^[^ ^9^ ^]^

In fast‐charging scenarios, high current densities drive massive Na⁺ migration toward the cathode surface, where the efficiency of Na⁺ crossing the CEI from the solvated state in the electrolyte into the cathode lattice becomes critical.^[^ ^10^ ^]^ Non‐ideal CEI layers, rich in high‐impedance organic/inorganic components, create significant kinetic barriers, leading to steep interfacial impedance and polarization, which restrict the maximum charging rate. Additionally, high potential and local Na⁺ concentration gradients exacerbate electrolyte oxidation, producing more resistive CEI components and accelerating layer thickening.^[^ ^11^ ^]^ Furthermore, mechanical stress from rapid sodium intercalation induces microcracks on cathode particles, exposing fresh active materials to direct contact with the electrolyte. This triggers uncontrolled side reactions, forming thicker, more uneven, and higher‐impedance non‐protective interfacial layers.^[^ ^12^ ^]^

Under wide‐temperature operations, interfacial challenges exhibit distinct but equally critical characteristics. At low temperatures, ion mobility plummets, and CEI formation kinetics, governed by a negative temperature coefficient, approach near‐insulating states, creating an insurmountable barrier for sodium‐ion transport.^[^ ^13^ ^]^ Simultaneously, interfacial charge transfer kinetics are severely suppressed, leading to exponential growth in interfacial impedance, manifesting as collapsed voltage plateaus and capacity cliffs. At high temperatures, thermodynamic forces accelerate electrolyte oxidation on high‐voltage cathode surfaces, causing disordered CEI growth—organic component decomposition and inorganic salt dissolution/recrystallization result in porous structures with plummeting ionic conductivity.^[^ ^14^ ^]^ More critically, the mechanical integrity of the CEI deteriorates at elevated temperatures, leading to cracking or delamination that exposes fresh cathode surfaces. This triggers intense electrolyte decomposition, active material consumption, gas evolution, and electrode structure destruction, accelerating capacity decay. Despite extensive research on electrolyte optimization, single‐electrolyte engineering cannot resolve the multidimensional challenges imposed by fast charging and extreme temperatures.^[^ ^15^, ^16^ ^]^ A significant gap persists in current studies: strategic design and control targeting the inherent interfacial characteristics of cathode materials—the microscopic foundations of CEI formation and function remain underexplored. The complex coupling mechanisms and microenvironment regulation at the CEI still represent an urgent scientific frontier that remains to be conquered.

Herein, we propose an innovative strategy utilizing 1,3‐propylene glycol (PDO) to perform hydroxylation modification on the surface of Na_3_V_2_(PO_4_)_3_ material (denoted as PDO40). The hydrogen‐bond network formed between surface hydroxyl groups and electrolyte molecules in PDO40 significantly promotes the formation of a stable CEI. The presence of surface hydroxyl groups has been confirmed by Fourier‐transform infrared spectroscopy (FT‐IR), X‐ray photoelectron spectroscopy (XPS), and thermogravimetric–mass spectrometry (TG‐MS). Density functional theory (DFT) calculations reveal that the hydrogen‐bond interaction between surface hydroxyls and the electrolyte specifically directs the decomposition pathway of fluorinated ethylene carbonate (FEC), preferentially generating a CEI layer enriched with stable NaF inorganic components. This CEI layer effectively suppresses parasitic reactions at the electrode interface, markedly mitigates volumetric strain during Na⁺ de‐/intercalation, and enhances sodium‐ion transport kinetics. Consequently, PDO40 exhibits exceptional cycling stability (72.84% capacity retention after 10 000 cycles at 10C) and outstanding wide‐temperature adaptability: at 80 °C and 10C rate, it maintains ultra‐high capacity retention over 1600 cycles with an average per‐cycle decay rate of merely 0.0099%; at −60 °C and 0.5C rate, it sustains a specific capacity of 66.13 mAh·g^−1^ without degradation over 1000 cycles. This surface hydroxylation strategy establishes a novel intrinsic interface engineering paradigm for developing fast‐charging, wide‐temperature cathode materials of SIBs.

## 2 Results and Discussion

**Figure 1:**
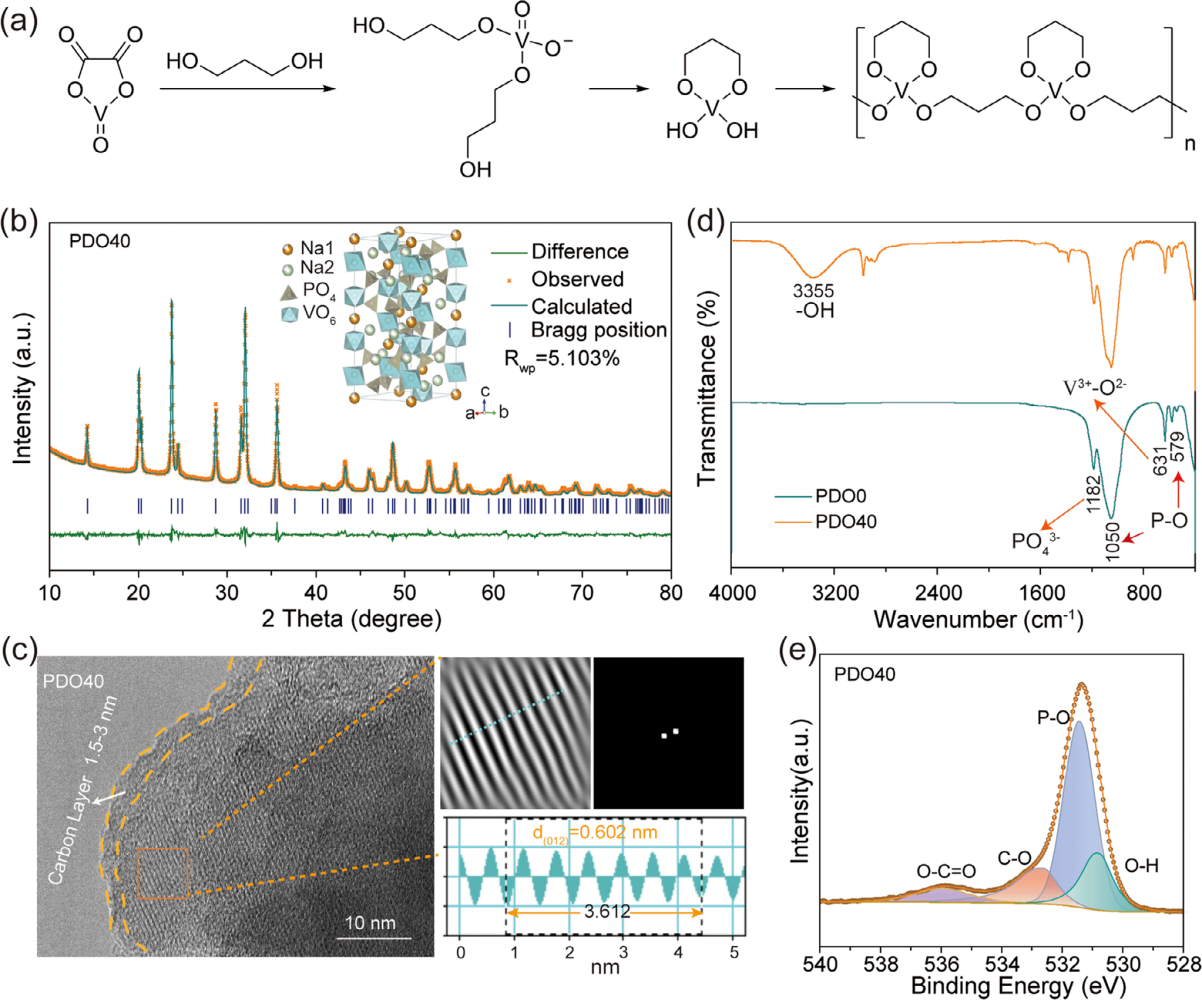
Mechanism of material synthesis, structural and morphological characterization. a) Possible reaction mechanisms of PDO40. b) XRD Rietveld refinement results of PDO40 (inset: the crystal structure). c) HR‐TEM image with the corresponding FFT pattern in the top‐right and an intensity line profile in the bottom‐right. d) FT‐IR spectra. e) High‐resolution XPS spectra of O 1s.

To elucidate the hydrogen‐bonding coupling mechanism proposed in our interfacial design strategy, **Figure** **1**a schematically outlines the synthesis process and possible structural evolution mechanism of PDO40. Initially, H_2_C_2_O_4_·2H_2_O, and V_2_O_5_ reacted to form vanadium oxalate (VOC_2_O_4_). Upon the addition of PDO, the oxalate group of VOC_2_O_4_ was gradually replaced to form vanadium propanolate. Subsequently, vanadium propanolate underwent protonation and oligomerization reactions to form vanadium‐diol complexes. Finally, the vanadium nuclei were connected by the abundant hydroxyl groups and the chelating ability of PDO.^[^ ^17^ ^]^ The cathode material without PDO is marked as PDO0. As shown in Figure S1 (Supporting Information), the X‐ray diffraction (XRD) peaks of both materials correspond well to the NASICON structure of Na_3_V_2_(PO_4_)_3_ (PDF #00‐62‐0345), and the introduction of PDO does not change the crystal structure. Furthermore, Rietveld refinement of XRD shows that the PDO0 and PDO40 both belong to the typical *R‐3c* rhombohedral crystal system (Figure 1b; Figure S2, Supporting Information). The detailed structural information is listed in Tables S1–S3 (Supporting Information). The morphology of PDO0 and PDO40 was examined using scanning electron microscopy (SEM) and high‐resolution transmission electron microscopy (HR‐TEM). SEM images (Figure S3, Supporting Information) reveal PDO40 possesses smaller primary particles than PDO0, along with a slightly higher Brunauer–Emmett–Teller (BET) surface area (30.26 vs 25.68 m^2^ g^−1^) and more uniform porosity (Figure S4, Supporting Information). The reduced particle size can shorten the Na^+^ diffusion pathway, and the enhanced surface area and pore structure provide greater contact area between the active material and electrolyte.^[^ ^18^ ^]^ Furthermore, from the HR‐TEM images (Figure 1c; Figure S5, Supporting Information), it can be observed that the surface of the materials was all encapsulated with a thin carbon layer, which helps to increase electronic conductivity and buffer volume changes.^[^ ^19^ ^]^ The clear lattice fringe spacings of 0.602 and 0.591 nm for PDO40 and PDO0, respectively, correspond to the (012) crystal plane. Moreover, the fast Fourier transform (FFT) patterns confirm the high crystallinity of both samples. SEM energy dispersive spectrometer (EDS) mappings (Figures S6 and S7, Supporting Information) clearly illustrate the homogeneous distribution of all elements (Na, V, P, O, C) within the prepared particles. FT‐IR is used to analyze the chemical bonding on the surface of the materials, as shown in Figure 1d, the bands at 1050 and 579 cm^−1^ are attributed to the asymmetric stretching vibration and the asymmetric bending vibration of P─O bonds in the PO_4_ tetrahedron, respectively. The band at 1182 cm^−1^ corresponds to another mode of the asymmetric stretching vibration of the PO_4_ unit, whereas the band at 631 cm^−1^ is associated with the bending vibration of the V^3+^─O^2−^ bond in the isolated VO_6_ octahedra.^[^ ^20^ ^]^ It should be noted that PDO40 reveals a characteristic hydroxyl stretching vibration at 3355 cm^−1^, indicating that the surface of PDO40 contains hydroxyl groups, which are capable of forming hydrogen bonds with electrolyte molecules to build a stable interfacial layer. Furthermore, the temperature‐dependent evolution of H_2_O was investigated using TG‐MS, as illustrated in Figure S8 (Supporting Information).^[^ ^21^ ^]^ The minor peaks observed below 200  °C are attributed to the evaporation of surface‐adsorbed water. Obviously, PDO40 manifests a pronounced enhancement in H_2_O release between 200 and 500 °C, corresponding to the decomposition of structural hydroxyl within the bulk material. The hydroxyl groups could act as hydrogen bond donors to electrolyte molecules, templating an ordered interfacial structure that enhances cathode stability. Conversely, the H_2_O release curve of PDO0 remains essentially unchanged across the same temperature range, indicating the absence of hydroxyl groups, which is consistent with the FT‐IR results. The surface elemental composition and valence states of the materials were further probed using XPS. As shown in Figure S9 (Supporting Information), the signals of each element are clearly distinguishable in the full‐scale XPS spectra of PDO40 and PDO0. In the high‐resolution O 1s spectra (Figure 1e; Figure S10, Supporting Information), the peaks at 531.46/531.36 eV, 532.71/532.59 eV, and 536.07/535.98 eV are assigned to P─O, C─O, and O─C═O bonds, respectively.^[^ ^22^ ^]^ Notably, there is an additional peak at 530.9 eV in PDO40, which belongs to ─OH, further confirming the presence of hydroxyl groups in the surface.^[^ ^23^ ^]^ The high‐resolution V 2p spectra (Figure S11, Supporting Information) demonstrate two peaks at 517.13 and 524.16 eV, corresponding to the V 2p_3/2_ and V 2p_1/2_ of V^3+^.^[^ ^24^, ^25^ ^]^ The thermogravimetric curves show a slight increase in carbon content due to the introduction of PDO (Figure S12, Supporting Information). Moreover, the Raman spectra of PDO0 and PDO40 (Figure S13, Supporting Information) feature distinct characteristic peaks corresponding to the disordered carbon (D band) and graphitic carbon (G band).^[^ ^26^ ^]^ The intensity ratio (*I_D_/I_G_*) of PDO40 (1.33) was marginally higher than that of PDO0 (1.19), indicating that the introduction of hydroxyl groups increases the defects in the carbon material.

### 2.1 Electrochemical Performance

**Figure 2:**
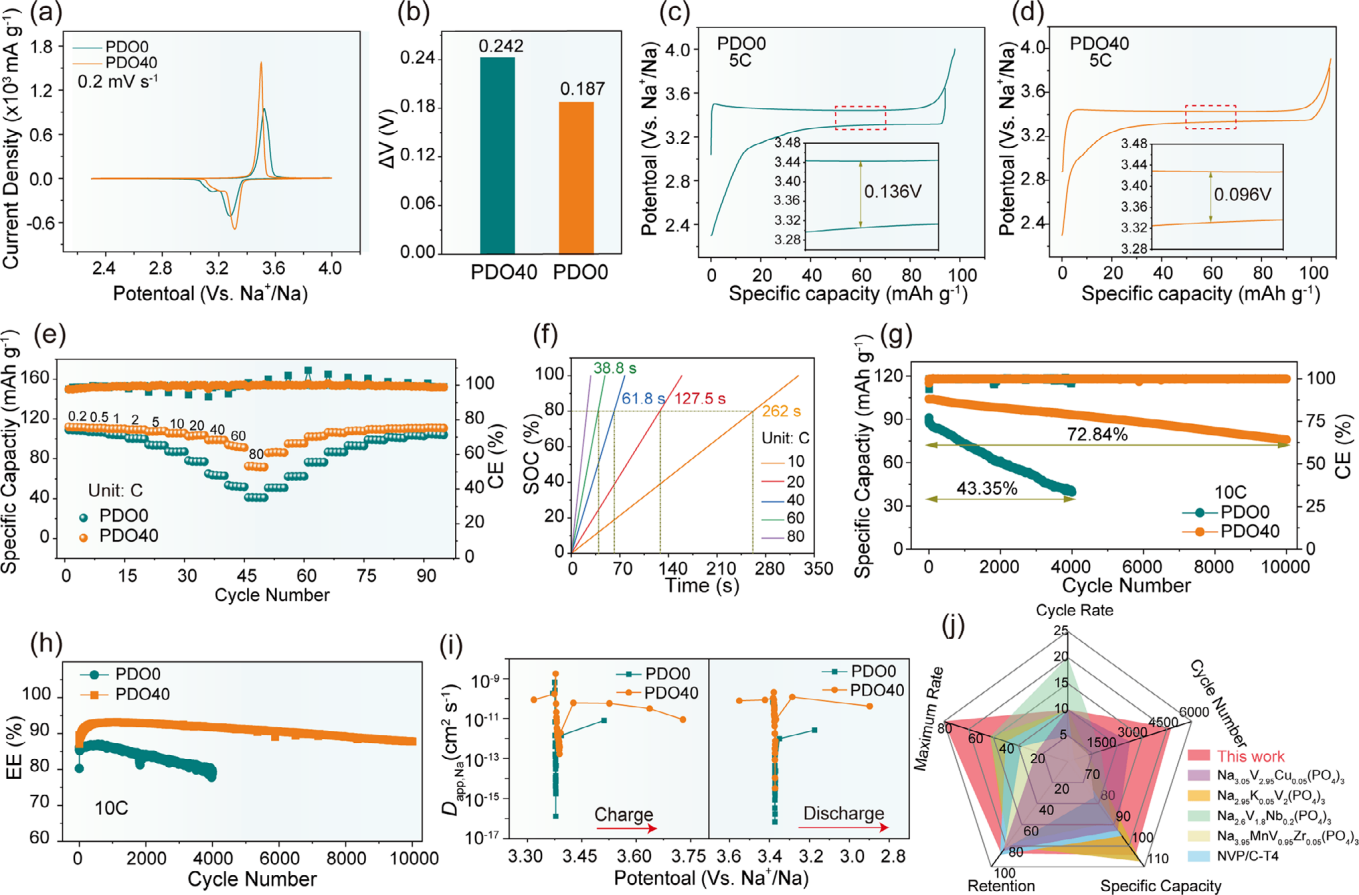
Electrochemical and kinetic properties at room temperature. a) CV curves at 0.2 mV s^−1^. b) ΔV from CV curves. GCD curves. c) PDO0. d) PDO40. e) Rate performance from 0.2C to 80C. f) SOC‐time of PDO40. g) Ultra‐long cycling performance at 10C. h) EE curves at 10C cycling. i) *D*_app,Na_ calculated from the GITT test. j). Comparison of the electrochemical performance of PDO40 and other phosphate cathode materials.

By establishing hydrogen‐bond‐coupling mechanisms between the hydroxyl groups on the material surface and the electrolyte molecules, the interface microenvironment was precisely regulated, leading to excellent electrochemical performance of PDO40 in half cells. As shown in the cyclic voltammetry (CV) curves of **Figure** **2**a, the oxidation peak at ≈3.5 V and the reduction peak at ≈3.3 V are attributed to the V^4+^/V^3+^ redox pair. It is evident that the PDO40 oxidation peak and reduction peak are sharper, along with a smaller polarization (ΔV) as shown in Figure 2b (0.187 vs 0.242 V). Correspondingly, the plateaus are also observed in the galvanostatic charge–discharge (GCD) curves in Figure 2c,d. At a current density of 5C, PDO40 and PDO0 deliver initial discharge specific capacities of 107.31 and 94.10 mAh g^−1^, respectively. Moreover, the GCD curves of PDO40 display minimal voltage hysteresis, demonstrating reduced polarization compared to PDO0 (0.096 vs 0.136 V), suggesting that PDO40 has better reversibility and faster kinetics. Additionally, PDO0 exhibits significantly more severe self‐discharge, attributed to unstable CEI that triggered side reactions at the interface. In contrast, PDO40 forms stable CEI due to surface hydroxyl groups, suppressing side reactions and markedly alleviating self‐discharge. Subsequently, the rate capability of the materials is systematically evaluated across a wide range of current densities (0.2C–80C). As shown in Figure 2e, compared to PDO0, PDO40 has a significant advantage in rate performance. Specifically, PDO40 achieves discharge specific capacities of 112.17, 110.8, 105.65, 102.56, 98.67, and 93.89 mAh g^−1^ at 0.2C, 0.5C, 10C, 20C, 40C, and 60C, respectively. Remarkably, even at an ultrahigh rate of 80C, PDO40 still retains a specific capacity of 72.65 mAh g^−1^, with each charge completed in just 27 s. Upon returning to 0.2C, PDO40 recovers 111.05 mAh g^−1^, corresponding to 99% of its initial capacity, demonstrating its exceptional reversibility. In contrast, PDO0 shows inferior rate capability, with a specific capacity of 109.1 mAh g^−1^ at 0.2C, which plummets to 41.2 mAh g^−1^ at 80C. When cycled back to 0.2C, PDO0 recovers just 96.33% of its capacity. Furthermore, PDO40 maintains 91.4% specific capacity retention at 20C and 64.76% at 80C, whereas PDO0 retains only 78.1% at 20C and 37.76% at 80C (Figure S14, Supporting Information). Interestingly, the coulombic efficiency (CE) of PDO40 remains stable at different rates, basically at 100%, while PDO0 shows obvious fluctuations, further demonstrating the stability of PDO40. GCD curves at different rates (Figure S15, Supporting Information) clearly show that as current density increases, the polarization of PDO40 is significantly lower than that of PDO0. Moreover, even at a high current density of 80C, PDO40 retains distinct charge–discharge plateaus. Additionally, PDO40 also delivers enhanced energy and power density compared to PDO0 (Figure S16, Supporting Information). Notably, the PDO40 cathode demonstrates exceptional fast‐charging performance, achieving 80% state of charge (SOC) in merely 38.8 s at an ultrahigh current rate of 60C (Figure 2f). Additionally, PDO40 maintains superior cycling stability. As shown in Figure S17 (Supporting Information), the PDO40 cathode delivers an initial specific capacity of 109.15 mAh g^−1^ at 1C while maintaining 94.93% capacity retention after 500 cycles (80.02% of PDO0). Surprisingly, PDO40 achieves an initial specific capacity of 104.42 mAh g^−1^ at a high rate of 10C, with outstanding capacity retention of 86.99% after 5000 cycles. Impressively, it maintains a reversible specific capacity of 75.88 mAh g^−1^ even after 10 000 cycles (Figure 2g). In stark contrast, the PDO0 cathode not only delivers a lower initial specific capacity (91.15 mAh g^−1^) but also suffers a rapid capacity decay, with only 43.35% retention after 4000 cycles at 10C. Furthermore, throughout the cycling process, PDO40 consistently exhibits more pronounced charge–discharge plateaus and lower polarization (Figure S18, Supporting Information). Figure 2h presents the energy density (EE) corresponding to long‐term cycling at 10C, the average EE of PDO40 reaches 90.90%, while PDO0 is only 83.73%. This significant difference demonstrates that PDO40 possesses superior energy conversion capability under high‐rate cycling, suggesting its promising potential for practical applications.

The excellent fast charging performance and cycle stability are attributed to the hydrogen bond coupling between the surface hydroxyl groups and the electrolyte molecules. Subsequently, the diffusion kinetics of Na^+^ were evaluated using multi‐scan rates CV and galvanostatic intermittent titration technique (GITT). Specifically, the multi‐scan CV curves in Figure S19 (Supporting Information) clearly show that PDO40 has lower polarization and higher peak intensity. In addition, the linear relationship between peak current and the square root of scan rate confirms diffusion‐controlled Na⁺ de‐/intercalation behavior.^[^ ^27^ ^]^ By calculating the Na⁺ diffusion coefficient (*D*_app,Na_) from CV, the value of PDO40 ($\bar{D}$_app,Na_ = 2.68 x 10^−9^ cm^2^ s^−1^) is one order of magnitude higher than that of PDO0 ($\bar{D}$_app,Na_ = 4.89 x 10^−10^ cm^2^ s^−1^) (Figure S20, Supporting Information). From the GITT test, Figure S21 (Supporting Information) shows the change in *D*_app,Na_ over time, and the single titration curve of PDO40. The *D*_app,Na_ at different charge states as shown in Figure 2i. It can be seen that the *D*_app,Na_ values of both PDO40 and PDO0 decrease sharply ≈3.4 V. This is due to a phase transition, which leads to slower Na^+^ diffusion within this voltage range, corresponding to the charge–discharge plateaus observed in the GITT curves (Figure S22, Supporting Information).^[^ ^19^ ^]^ It is worth noting that during the entire charge–discharge process, the *D*_app,Na_ values of PDO40 ($\bar{D}$_app,Na_ = 2.38 x 10^−10^ cm^2^ s^−1^) is significantly higher than those of PDO0 ($\bar{D}$_app,Na_ = 1.71 x 10^−11^ cm^2^ s^−1^), enabling faster Na^+^ diffusion. Additionally, the Nyquist plot of the electrochemical impedance and the equivalent circuit diagram corresponding to the fitted data are shown in Figure S23 (Supporting Information). Here, *Rs* represents the solution resistance, *R1* corresponds to the interfacial impedance of the CEI, *Rct* (typically corresponding to the semicircle in the high‐frequency region) denotes the charge transfer resistance, and *Zw* represents the diffusion impedance.^[^ ^28^ ^]^ It can be clearly observed that the *Rct* of PDO40 is significantly smaller than that of PDO0 (137 vs 197 Ω). These collective findings establish PDO40 as a superior cathode material with optimized ion diffusion pathways and improved charge transfer characteristics. Compared with reported phosphate cathode materials, the PDO40 exhibits outstanding performance in all aspects (Figure 2j).^[^ ^29^, ^30^, ^31^, ^32^, ^33^ ^]^

**Figure 3:**
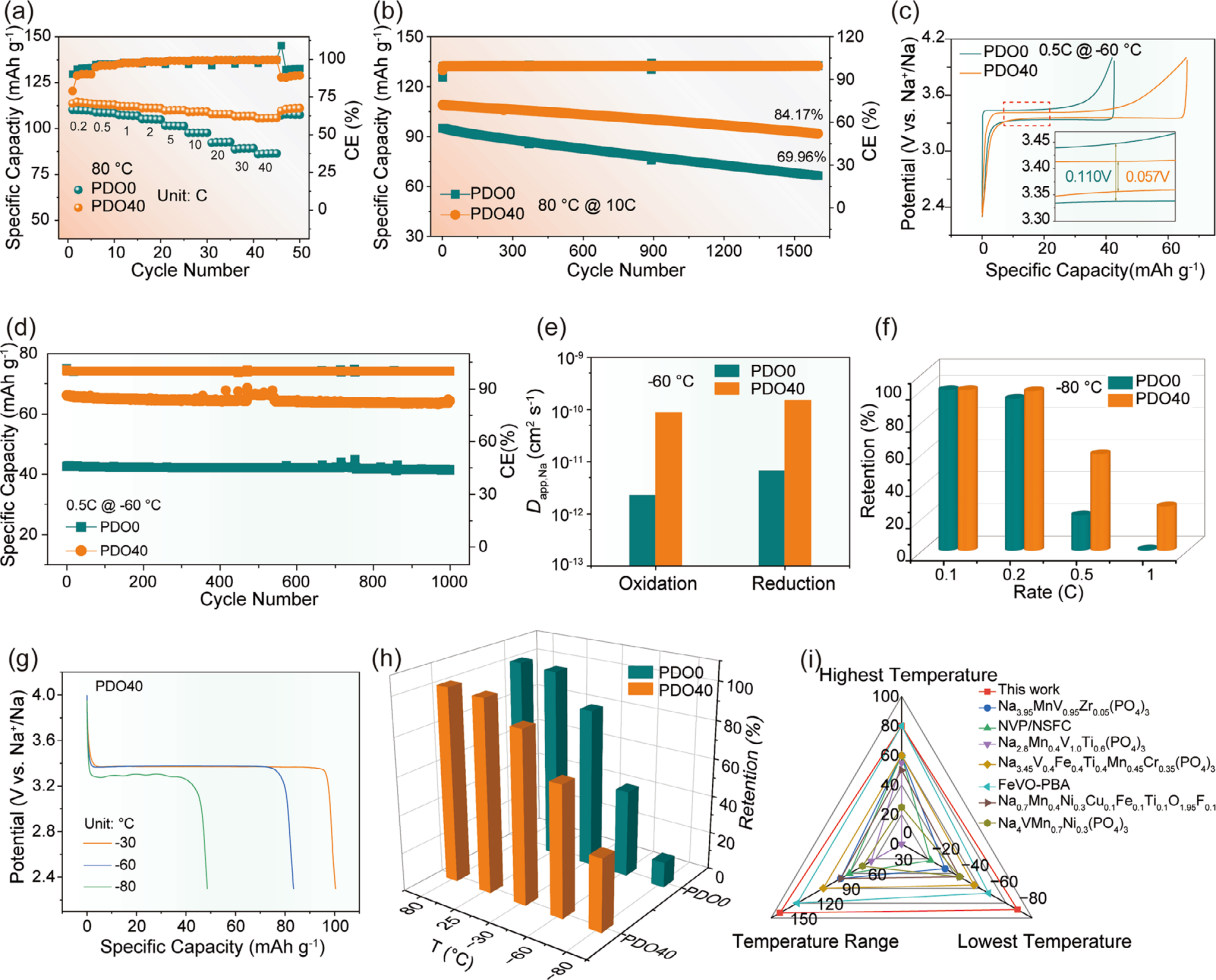
Electrochemical and kinetic performance at high and low temperatures. a) Rate performance at 80 °C. b) Cycling performance at 10C at 80 °C. c) GCD curves at 0.5C at −60 °C. d) Cycling performance at 0.5C at −60 °C (e) *D*_app,Na_ calculated from CV test. f) Capacity retention at different rates at −80 °C. g) Discharge curves at 0.1C under various low‐temperature conditions. h) Capacity retention at different temperatures at 0.2C. i) Comparative temperature range of PDO40 with reported wide‐temperature studies.

The surface hydroxyl groups serve as a universal interface regulator across wide temperature ranges, directing the formation of an adaptive CEI layer that enables exceptional electrochemical performance under extreme temperatures. At high‐temperatures, the enhancement in ion migration kinetics leads to improved electrochemical performance compared to room‐temperature. Specifically, at 80 °C, the initial discharge specific capacities of PDO40 and PDO0 at 0.2C are 113.71 and 110.34 mAh g^−1^, respectively. Regardless of current density, PDO40 consistently outperformed PDO0, maintaining a discharge specific capacity of 105.64 mAh g^−1^ even at 40C (**Figure** **3**a). A critical challenge for high‐temperature electrode materials lies in ensuring long‐term cycling stability. The presence of hydroxyl groups facilitates the formation of a stable CEI membrane, enabling PDO40 to sustain stable high‐rate cycling. Remarkably, after 1600 cycles at 10C, PDO40 retains 84.17% of its initial specific capacity (109.04 mAh g^−1^), while PDO0 only maintains a capacity retention of 69.96%, with its capacity decaying to 66.53 mAh g^−1^ (Figure 3b). This demonstrates the exceptional fast‐charging cycle stability of PDO40. In addition, Figure S24 (Supporting Information) shows superior EE at high temperature cycling. The performances at low‐temperatures are systematically evaluated. As shown in Figure S25 (Supporting Information), in the first‐cycle discharge at −30 °C, PDO40 provides a specific capacity of 101 mAh g^−1^ in both ester‐based (PC) and ether‐based (G2) electrolytes, significantly outperforming PDO0. However, it can be clearly seen that both materials display obvious voltage oscillations in the PC electrolyte, while the ether‐based electrolyte perfectly resolves this issue, which is consistent with the literature reports.^[^ ^34^ ^]^ Consequently, all subsequent low‐temperature data are obtained using G2 electrolyte. Furthermore, the discharge curves acquired confirm that PDO40 attains 86.35 mAh g^−1^ at 1C and 69.83 mAh g^−1^ at 5C, whereas PDO0 yields only 60.66 mAh g^−1^ at 1C and 49.56 mAh g^−1^ at 5C, along with increased polarization (Figure S26, Supporting Information). This demonstrates the superior potential of PDO40 for low‐temperature fast charging applications. Subsequently, Figure 3c shows the GCD curves at −60 °C, the PDO40 voltage hysteresis is smaller, suggesting that Na^+^ has faster kinetics in the de‐/intercalation process. Consequently, the initial specific capacity at 0.5C surpasses 55% than PDO0 (66.13 vs 42.61 mAh g^−1^), and maintains ≈100% capacity retention after 1000 cycles (Figure 3d). These findings are further corroborated by CV at various scan rates and calculated *D*_app,Na_ (Figure 3e; Figure S27, Supporting Information). Remarkably, even at 60 °C, PDO40 exhibits a high *D* *_app,Na_* of 1.17 x 10^−10^ cm^2^ s^−1^, significantly surpassing PDO0 by over an order of magnitude, confirming that the enhanced ionic diffusion constitutes the fundamental mechanism for superior capacity of PDO40 at low‐temperature. Even at an ultra‐low temperature of −80 °C, there is still a specific capacity of 48.6 mAh g^−1^ of PDO40 at 0.1C, and it has better rate performance and stability than PDO0 (Figure 3f; Figure S28, Supporting Information). Meanwhile, this hydrogen‐bond regulated interface enables the material to maintain well‐defined discharge plateaus even at different low temperatures, while PDO0 shows shorter plateaus and greater polarization due to its lack of hydroxyl groups. (Figure 3g; Figure S29, Supporting Information). Consequently, it makes the PDO40 cathode shows exceptional capacity retention across an ultra‐wide temperature range (−80–80 °C, Figure 3h), demonstrating superior wide‐temperature adaptability compared to other cathode materials (Figure 3i).^[^ ^4^, ^31^, ^35^, ^36^, ^37^, ^38^, ^39^ ^]^

### 2.2 Hydrogen Bond Coupling Mechanism

**Figure 4:**
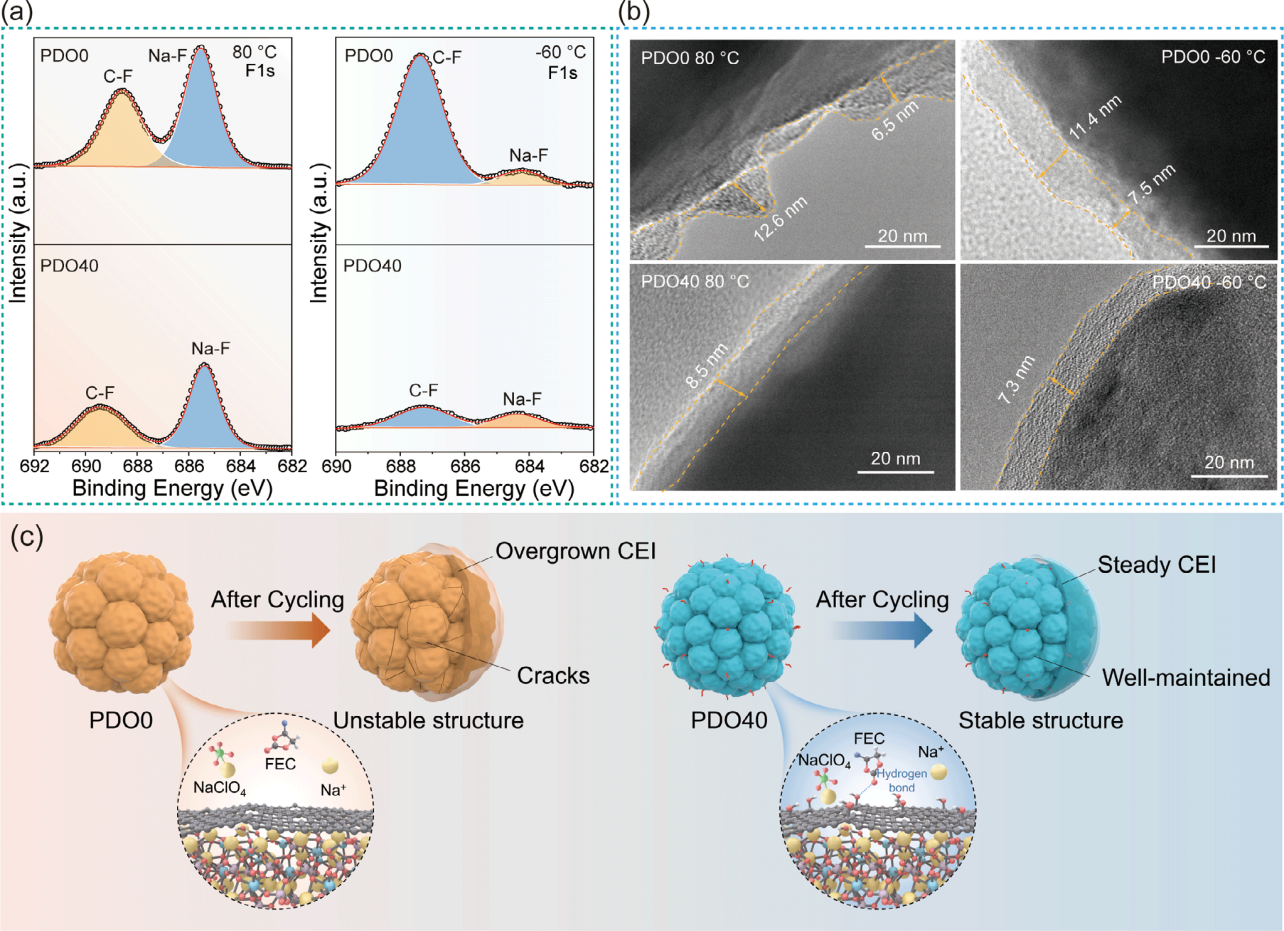
Analysis of CEI Layer characteristics. a) XPS spectra of F 1s of PDO0 and PDO40 after cycling at different temperatures. b) TEM images of PDO0 and PDO40 after cycling at different temperatures. c) Schematic diagram of the structural stability of PDO0 and PDO40 particles after cycling.

The morphological and structural characterization after the cycle further revealed the role of hydroxyl groups in regulating the interface microenvironment. The CEI composition of the cycled electrode was analyzed by XPS (**Figure** **4**a; Figure S30, Supporting Information). The F 1s spectra of XPS exhibit two characteristic peaks corresponding to Na─F (from CEI) and C─F (from the PVDF binder) at both temperature extremes, while the O 1s spectra reveal contributions from C─O/C═O, Na_2_CO_3_, and the Auger peak of sodium.^[^ ^40^, ^41^ ^]^ Notably, at 80 °C, the Na─F and Na_2_CO_3_ signals in PDO0 are significantly stronger, indicating more severe electrolyte decomposition on PDO0. However, due to the action of surface hydroxyl groups, PDO40 effectively mitigates further electrolyte decomposition. At −60 °C, the Na─F and Na_2_CO_3_ signals are markedly weaker than at 80 °C, and the proportion of NaF in PDO40 is significantly higher. This is primarily due to the hydrogen bonding between hydroxyl groups and electrolyte molecules, which promotes the formation of a stable CEI rich in NaF.^[^ ^42^, ^43^ ^]^ The role of surface hydroxyl functional groups enables PDO40 to suppress decomposition at high temperatures and promote uniform CEI film formation at low temperatures, which is key to its outstanding wide‐temperature performance. Furthermore, the TEM images in Figure 4b clearly show that the CEI of the PDO electrode is thick and uneven. Conversely, PDO40 is uniformly coated with a thin CEI layer at both high and low temperatures. This thinner CEI reduces the diffusion distance for sodium ions, thereby enhancing rate performance. A more uniform CEI composition minimizes electrolyte decomposition, maintains structural integrity during prolonged cycling, and facilitates efficient Na⁺ transfer at the interface.^[^ ^44^ ^]^ Consequently, PDO40 demonstrates outstanding rate capability and cycling stability across an ultra‐wide temperature range. Furthermore, XRD patterns reveal that the PDO40 electrode retains a well‐defined Na_3_V_2_(PO_4_)_3_ structure even after cycling at 25 and 80 °C. Conversely, PDO0 shows weaker diffraction peaks, with the peak at 14.3° almost disappearing completely, which confirms the structural degradation (Figure S31, Supporting Information). Additionally, SEM images (Figure S32, Supporting Information) describe that the PDO40 electrode maintains structural integrity after cycling at both room and high temperatures, exhibiting no obvious cracks. However, the PDO0 electrode displays obvious cracks, with a crack width of up to ≈6 nm after cycling at 80 °C, thereby resulting in poor cycling stability. More importantly, these comprehensive results demonstrate different structural evolution of PDO40 and PDO0 during electrochemical cycling, as schematically illustrated in Figure 4c. The hydroxyl enabled hydrogen bonding in PDO40 directs the formation of a robust and uniform CEI layer, capably maintaining electrode structural integrity and explaining its superior cycling stability compared to PDO0. The interaction between the hydroxyl group and the electrolyte will be discussed later.

**Figure 5:**
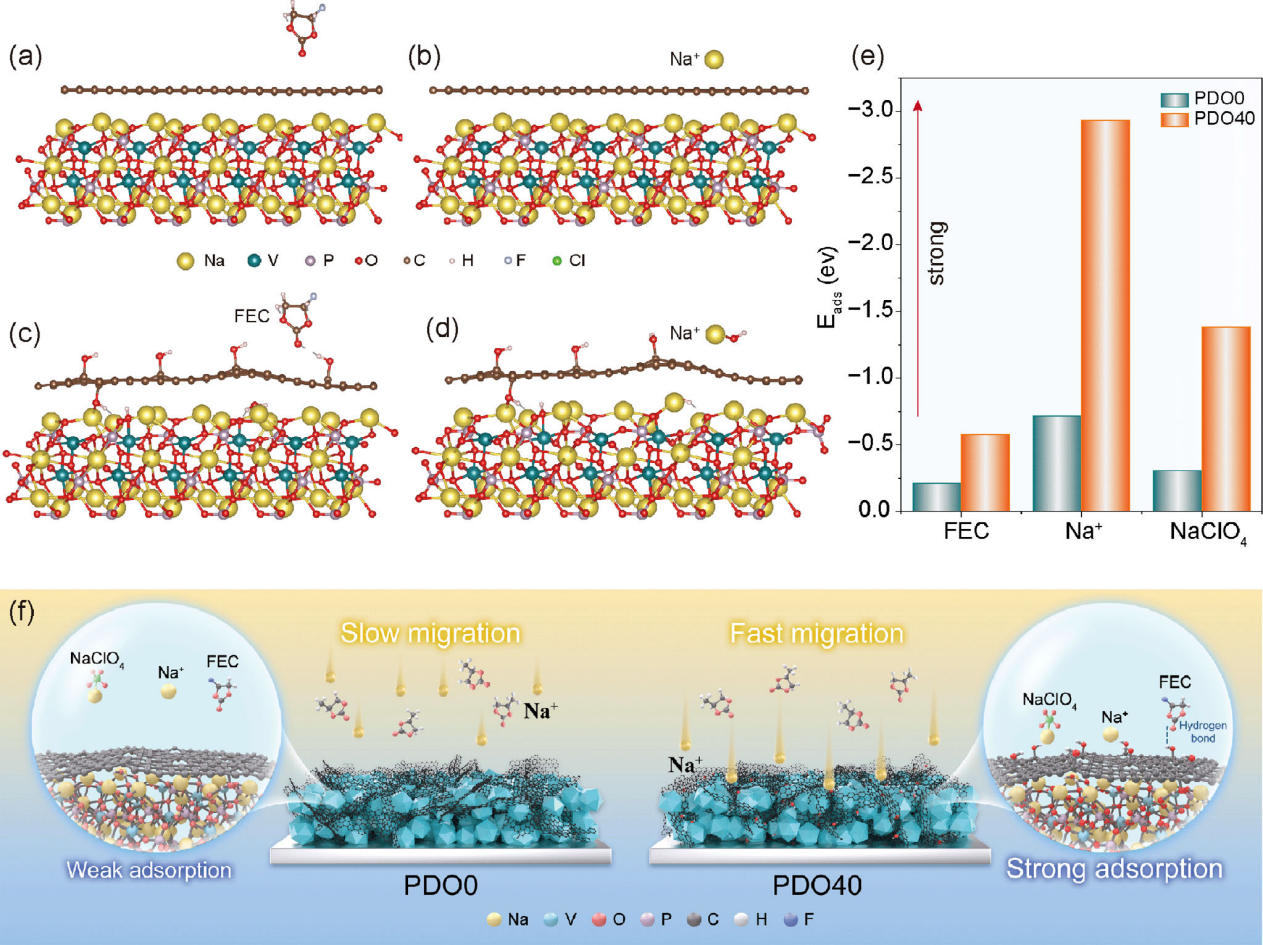
DFT calculations. Structural modeling of the after adsorption of FEC and Na^+^. a,b) PDO0. c,d) PDO40. e) E_ads_ with different molecules. f) Schematic diagram of Na^+^ de‐/intercalation mechanism.

DFT calculations further reveal that hydrogen‐bond coupling between surface hydroxyl groups and electrolyte molecules effectively modulates the interfacial microenvironment, driving the formation of a robust CEI layer. The optimized structural models of PDO40 and PDO0, along with their corresponding adsorption configurations of FEC, Na⁺, and NaClO_4,_ are illustrated in **Figure** **5**a–d and Figures S33 and S34 (Supporting Information), respectively. Notably, PDO40 exhibits stronger adsorption energy (E_ads_) toward these species compared to PDO0 (Figure 5e). As schematically depicted in Figure 5f, during the charge–discharge process, the absence of surface hydroxyl groups in PDO0, results in weak interactions with electrolyte components and sluggish Na⁺ migration kinetics. In contrast, the hydroxyl groups on the surface of PDO40 facilitate hydrogen bonding with FEC molecules, promoting their preferential decomposition to form a stable, NaF‐rich CEI layer. Most notably, the enhanced adsorption of NaClO_4_ effectively suppresses parasitic side reactions, while the stronger interaction with Na⁺ significantly lowers the interfacial diffusion energy barrier.^[^ ^45^ ^]^ These synergistic effects collectively accelerate Na⁺ migration kinetics, thereby enhancing the overall electrochemical performance of PDO40.

**Figure 6:**
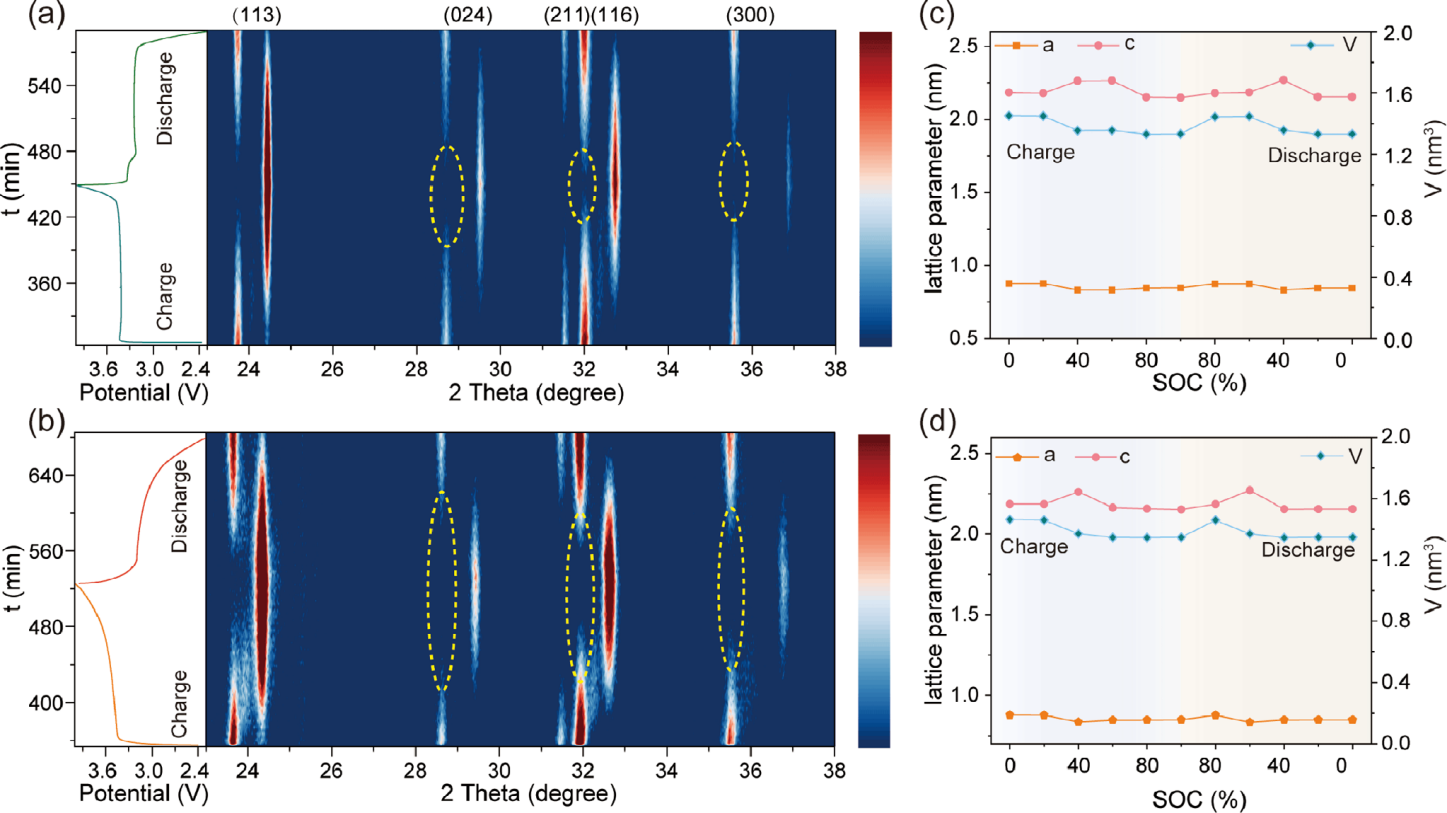
Structural evolution during charging and discharging. Contour maps of in situ XRD and corresponding GCD curves. a) PDO0. b) PDO40. Change of the lattice parameters from in situ XRD. c) PDO0. d) PDO40.

To elucidate how hydrogen‐bond coupling stabilizes the framework, we used in situ XRD to track the structural evolution of the material during Na⁺ de‐/intercalation. As shown in **Figure** **6**a,b, both materials follow the same phase transition. During charging, Na⁺ extraction from the crystal lattice, and the intensity of the diffraction peaks corresponding to the Na_3_V_2_(PO_4_)_3_ crystal planes (113), (024), (211), (116), and (300) gradually decreases until they disappear. Simultaneously, new diffraction peaks appear at higher diffraction angles, corresponding to the two‐phase reaction, and these new peaks are attributed to the NaV(PO_4_)_3_ structure.^[^ ^46^ ^]^ The discharge process is the opposite, with the NaV(PO_4_)_3_ phase gradually disappearing while the Na_3_V_2_(PO_4_)_3_ phase reappears, until all diffraction peaks return to their original positions at the end of discharge, indicating good reversibility. Unlike PDO0, the NaV(PO_4_)_3_ phase diffraction peak intensity of PDO40 is stronger, and during the phase transition, the coexistence of the Na_3_V_2_(PO_4_)_3_‐NaV(PO_4_)_3_ two‐phase system transitions to a more isolated state (as shown in the yellow‐marked area), indicating better crystal structural stability.^[^ ^47^ ^]^ Subsequently, the lattice parameter variations of PDO40 and PDO0 at different SOC were calculated to analyze the structural volume changes during charge/discharge processes, as depicted in Figure 6c,d. Despite the similar change patterns in both materials, the PDO40 experiences a volume change of 7.92%, which is less than PDO0 (8.21%). The smaller volume change is mainly due to hydrogen bonding interactions, which promote the formation of a more uniform and stable CEI during charging and discharging, thereby causing smaller lattice strain during Na^+^ de‐/intercalation.

### 2.3 Practical Application of the Cathode

**Figure 7:**
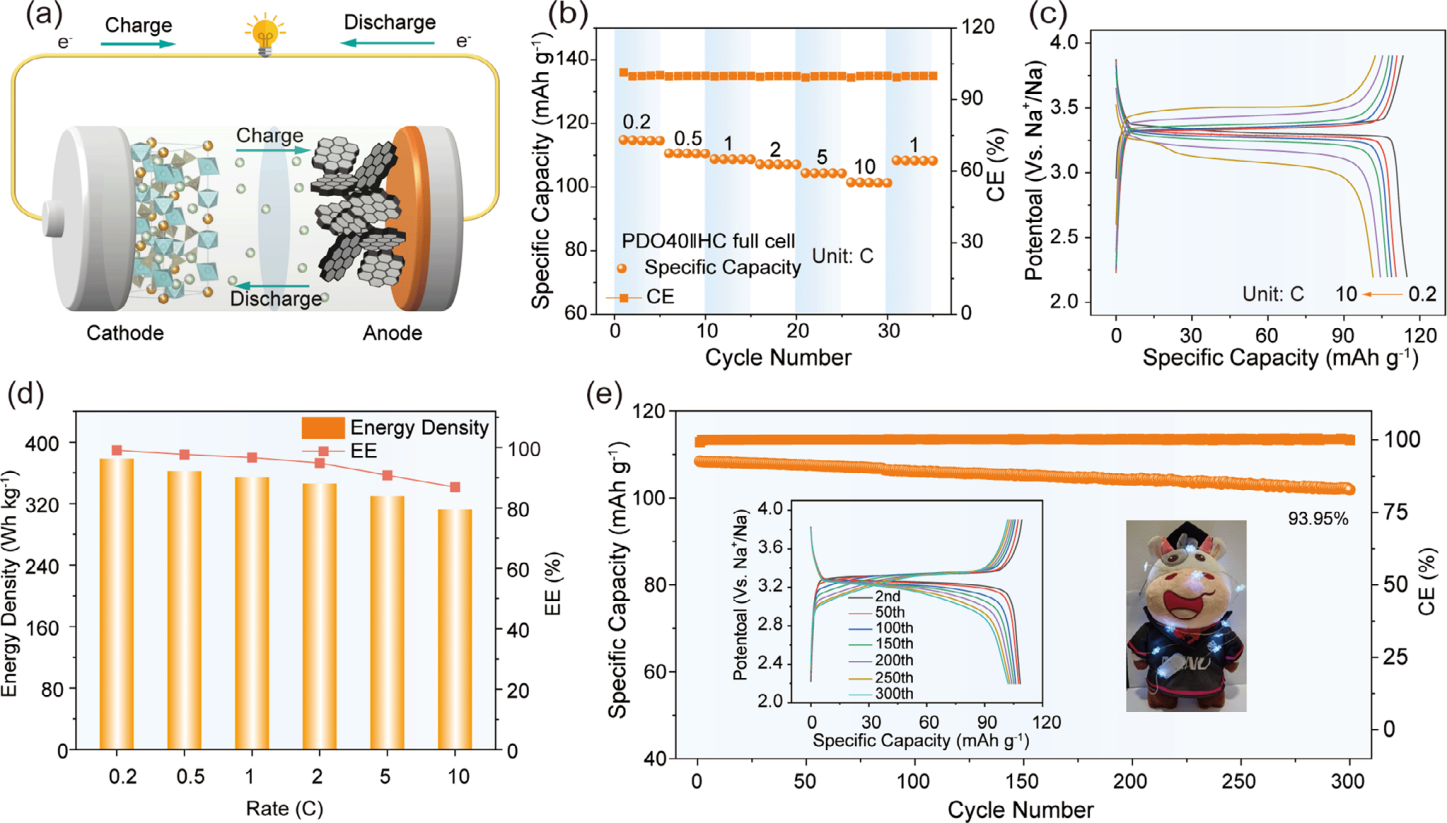
Performance of the PDO40||HC full cell. a) Schematic diagram of the full battery operation principle. b) Rate performance of the PDO40||HC full cell. c) Corresponding GCD curves at various rates. d) Energy density and EE of the full cell at different rates. e) Cycling performance of the full cell at 1C (insert: GCD curves for different cycles and the LED lights powered by the full cell).

Finally, full cells paired with commercial hard carbon (HC) anodes were assembled to assess the practicality of PDO40, and the working mechanism of the full cell is schematically illustrated in **Figure** **7**a. The characterization and electrochemical performance of HC are shown in Figures S35–S37 (Supporting Information). The charge specific capacity of the HC half‐cell is approximately equal to 300 mAh g^−1^ at 0.1C, confirming its suitability as an anode material. The full cell demonstrates exceptional rate performance (Figure 7b), achieving an initial specific capacity of 114.87 mAh g^−1^ at 0.2C. Even at 10C, it maintains a specific capacity of 101.51 mAh g^−1^. Furthermore, the minimal polarization observed in the GCD curves (Figure 7c) suggests rapid charge transfer and Na⁺ diffusion kinetics. In addition, the energy density of the full cell is as high as 378.2 Wh kg^−1^ at 0.2C, and the energy efficiency exceeds 80% at different rates (Figure 7d, based on the cathode mass). Moreover, the full cell exhibits outstanding cycling stability, retaining 93.95% of its initial capacity after 300 cycles at 1C (Figure 7e). The well‐maintained plateaus in the GCD curves (inset of Figure 7e) further confirm the outstanding stability and reversibility of the full cell. Notably, the practical applicability is evidenced by the successful operation of LEDs powered by the full cell, highlighting its significant potential for energy storage applications (insert of Figure 7e).

## 3 Conclusion

In summary, by leveraging hydroxyl‐mediated hydrogen bonding networks, we precisely control the interfacial microenvironment to achieve the formation of a uniform, thin, and stable CEI layer. Experiments combined with DFT calculations further reveal the mechanism by which the hydrogen bonding coupling between surface hydroxyl groups and electrolyte molecules contributes to the formation of stable CEI. This work breaks through the conventional approach to electrolyte engineering. The strategy validates the excellent performance of the Na_3_V_2_(PO_4_)_3_ cathode over an ultra‐wide temperature range. Specifically, PDO40 achieves 80% SOC in just 38.8 s at room temperature, an initial specific capacity of up to 109.04 mAh g^−1^ at 10C and 80 °C with a charging time of less than 6 min per cycle, and maintains 48.6 mAh g^−1^ at 0.1C even under −80 °C. This work provides a new way to develop energy storage systems with both ultra‐fast charging capability and extreme temperature adaptability.

## Conflict of Interest

The authors declare no conflict of interest.

## Supporting information



Supporting Information

## Data Availability

The data that support the findings of this study are available from the corresponding author upon reasonable request.
