# Supplementary material for: Hydrogen‐Bond‐Coupling Interfacial Microenvironment Enables Fast Charging of Sodium‐Ion Batteries Over a Wide Temperature Range
Source: Adv Sci (Weinh). 2025 Oct 27;13(1):e17001. doi: 10.1002/advs.202517001 (PMC12767094; doi:10.1002/advs.202517001)
Supplement: Supplementary file 1 — Supporting Information [file ADVS-13-e17001-s001.docx]

Supporting Information

Hydrogen-Bond-Coupling Interfacial Microenvironment Enables Fast Charging of Sodium-Ion Batteries over a Wide Temperature Range

*Jin-Ling Liu, Xiao-Tong Wang,* *Denglong Chen,^*^* *Zhen-Yi Gu,^*^ Yi-Fei Liu, Yan Zhuang, Yong-Li Heng, Hang Li, and Xing-Long Wu^*^*

Experimental Section

**Material Preparation**

The PDO40 cathode material was synthesized via a co-precipitation approach. Initially, a mixture containing 4 mmol of V_2_O_5_, 12 mmol of H_2_C_2_O_4_·2H₂O, and 15 ml of deionized water was prepared in a round - bottomed flask. This mixture was stirred at 70 °C for 30 min, after which 40 ml of 1,3-propanediol (PDO) was introduced and vigorously stirred for 4 h. Subsequently, a solution of 12 mmol of anhydrous NaH_2_PO_4_ and 0.3423 g of sucrose dissolved in 5 ml of deionized water was slowly added to the reaction mixture, and the reaction was continued for 15 min. After this addition, 40 ml of n-propanol was added dropwise, and the solution was stirred continuously for 1 h. The resulting solution was then dried by increasing the temperature to 150 °C. After grinding, the precursor was pre-calcined at 400 °C for 4 h under Ar2 atmosphere and then calcined at 800 °C for 8 h to obtain C-coated nano-Na_3_V_2_(PO_4_)_3_ material, labeled as PDO40. For comparison, PDO0 samples were prepared using the identical procedure but without PDO.

**Material characterizations**

X-ray diffraction (XRD) patterns were collected using a diffractometer (Bruker D8 Advance, Germany) with Cu Kα radiation over a 2θ range of 10-80°. Fourier-transform infrared spectroscopy (FT-IR) measurements were performed on a spectrometer (Thermo Nicolet IS 5, USA) to analyze the chemical bonds of the materials. Prior to testing, the samples were dried in vacuum at 120 °C for 24 h and then immediately pressed into pellets with KBr for FT-IR analysis. The morphology of the materials was examined by scanning electron microscopy (SEM, Hitachi SU 8000) and transmission electron microscopy (TEM, JEOL-2100F). X-ray photoelectron spectroscopy (XPS) was conducted using an electron spectrometer (VG Scientific ESCALab 220 i-XL) with Al Kα radiation (300 W) to determine the valence states of the elements in the materials. Thermogravimetric (TG) was performed on an analyzer (TG-DSC, STA409C) in the air atmosphere. Raman spectra were obtained using a Raman spectrometer (LabRAM HR800). The specific surface area was calculated by the Brunauer-Emmett-Teller (BET) method, and the average pore size was estimated using the Barrett-Joyner-Halenda (BJH) model. A thermogravimetric-mass spectrometry (TG-MS) system (Netzsch STA409PC and QMS403C) was employed to analyze the functional groups decomposed at various temperatures under N_2_ atmosphere. *In-situ* XRD patterns were collected during cycling using a custom cell that employed a beryllium plate simultaneously as the X-ray window and current collector, with galvanostatic tests conducted at 0.3 C.

**Electrochemical measurements**

The active material (PDO40 or PDO0) was mixed with Super P-Li and polyvinylidene fluoride (PVDF) at a ratio of 8:1:1. Then, N-Methylpyrrolidone (NMP) was added and the mixture was stirred homogeneously. The slurry was uniformly coated onto aluminum foils to form the electrodes. The electrodes were dried for 12 hours in a vacuum oven at 100 °C. Then, the electrode was cut into 12 mm diameter pieces. The active material loading amount for each electrode ranged from 1.2 to 1.6 mg.

C2032 button batteries were assembled in a glove box with a water and oxygen content of less than 0.1 ppm. Metal sodium was used as the counter and reference electrodes, Whatman glass fiber was used as the separator, and the electrolyte was 1 M NaClO₄ in propylene carbonate (PC) with 5 vol% fluoroethylene carbonate (FEC). The electrolyte at temperatures below -30°C was 0.8M NaPF6 in the diethylene glycol dimethyl ether and 1,3-dioxolane solution with a volume ratio of 8:2.

The battery was tested using galvanostatic charge-discharge (GCD) on a battery testing system (LAND CT2001) with a voltage window of 2.3-4 V versus Na⁺/Na. Cyclic voltammetry (CV) was tested using a CHI660 electrochemical workstation. The current density for the galvanostatic intermittent titration technique (GITT) test was 0.1C, with a single drop charging time of 30 minutes and a relaxation time of 3 hours. The electrochemical impedance (EIS, Princeton Applied Research P2000) was tested at a frequency of 0.01-106 Hz with a perturbation amplitude of 10 mV.

Hard carbon (HC) anode was composed of HC, C45, and sodium carboxymethyl cellulose (CMC) in a mass ratio of 8:1:1, with HC anode voltage window ranging from 0.01 to 3.0 V. The full cell was assembled using the PDO40 cathode and HC anode, with a voltage window of 2.2-3.9 V. The specific capacity of the full cell was determined based on the mass of cathode. To achieve a matched capacity between the cathode and anode, the capacity ratio of the HC anode to the PDO40 cathode was approximately 1.6:1 (the mass ratio is about 1:1.7). Additionally, the electrolyte used in both the HC half-cell and the full cell is 1M NaClO₄ in PC with 5% FEC. Prior to assembling the full cell, pre-sodiation the HC anode using the contact method. Specifically, 60 µL of electrolyte was dripped onto the surface of sodium metal. The HC anode was then placed over the sodium metal plate, and a flat object was used to apply light pressure for several seconds to ensure complete contact between the sodium metal and the HC anode. After maintaining this state for 10 hours, the pre-sodiated HC anode was assembled with the PDO40 cathode into a full cell.

**Computational Details**

All the calculations are performed in the framework of the density functional theory with the projector augmented plane-wave method, as implemented in the Vienna ab initio simulation package.^[1]^ The generalzied gradient approximation proposed by Perdew, Burke, and Ernzerhof is selected for the exchange-correlation potential.^[2]^ The cut-off energy for plane wave is set to 400 eV. The energy criterion is set to 10−5 eV in iterative solution of the Kohn-Sham equation. The Brillouin zone integration is performed using a 2x1x1 k-mesh. The DFT-D3 method was used to correct the van der Waals force. All the structures are relaxed until the residual forces on the atoms have declined to less than 0.05 eV/Å.

The adsorption energy (Eads) was calculated as:

E_ads_ = E_total_- E_slab_-E_adsorbate_

where E_total_ is the total energy of the optimized system, E_slab_ is the total energy of the clean surface slab, and E_adsorbate_ is the total energy of the isolated adsorbate.

Results and Discussion


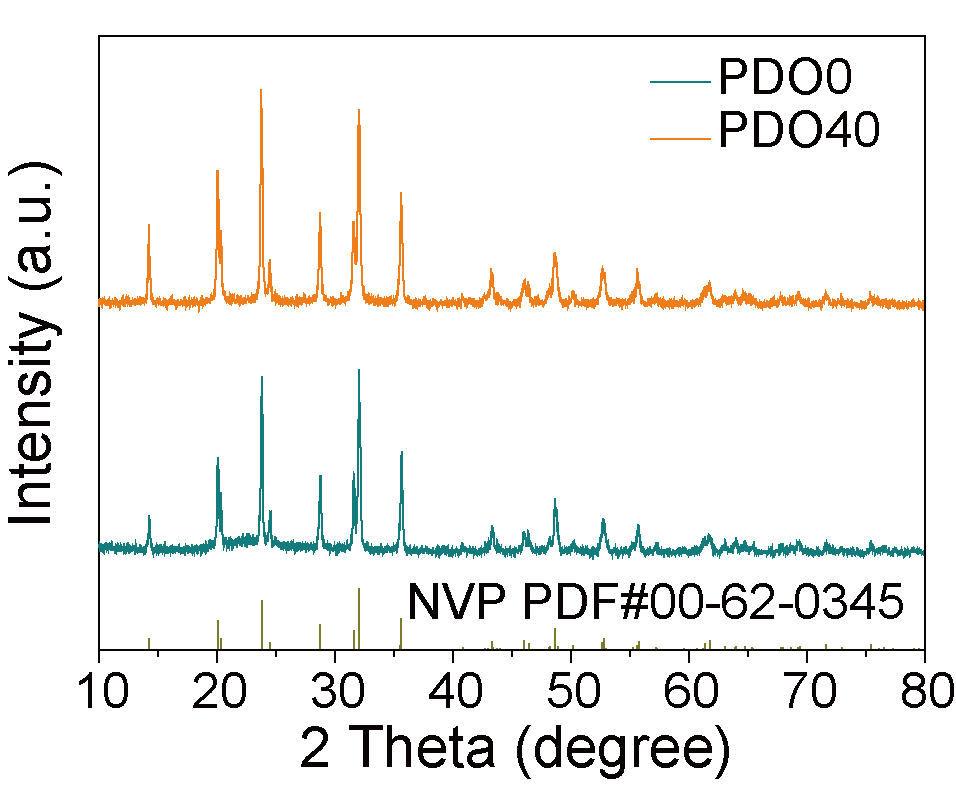


1. XRD patterns of powder samples.


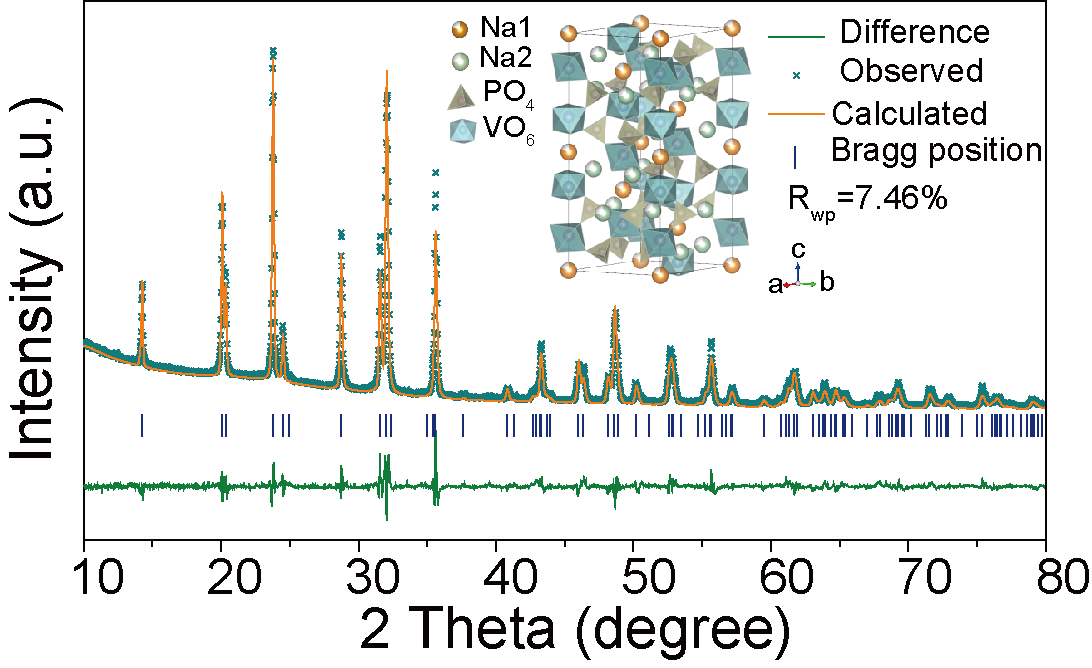


1. XRD rietveld refinement result of PDO0 (inset: the crystal structure).


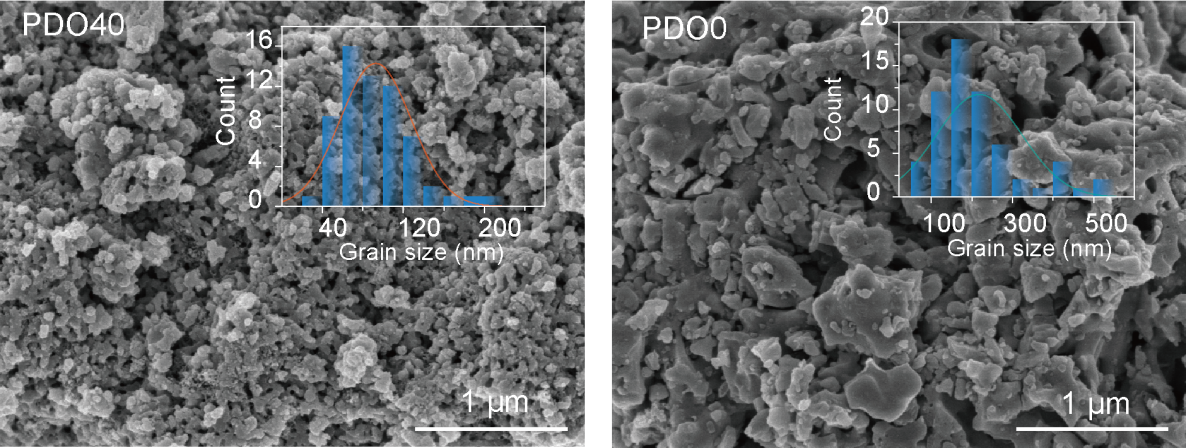


1. SEM images of PDO40 and PDO0.


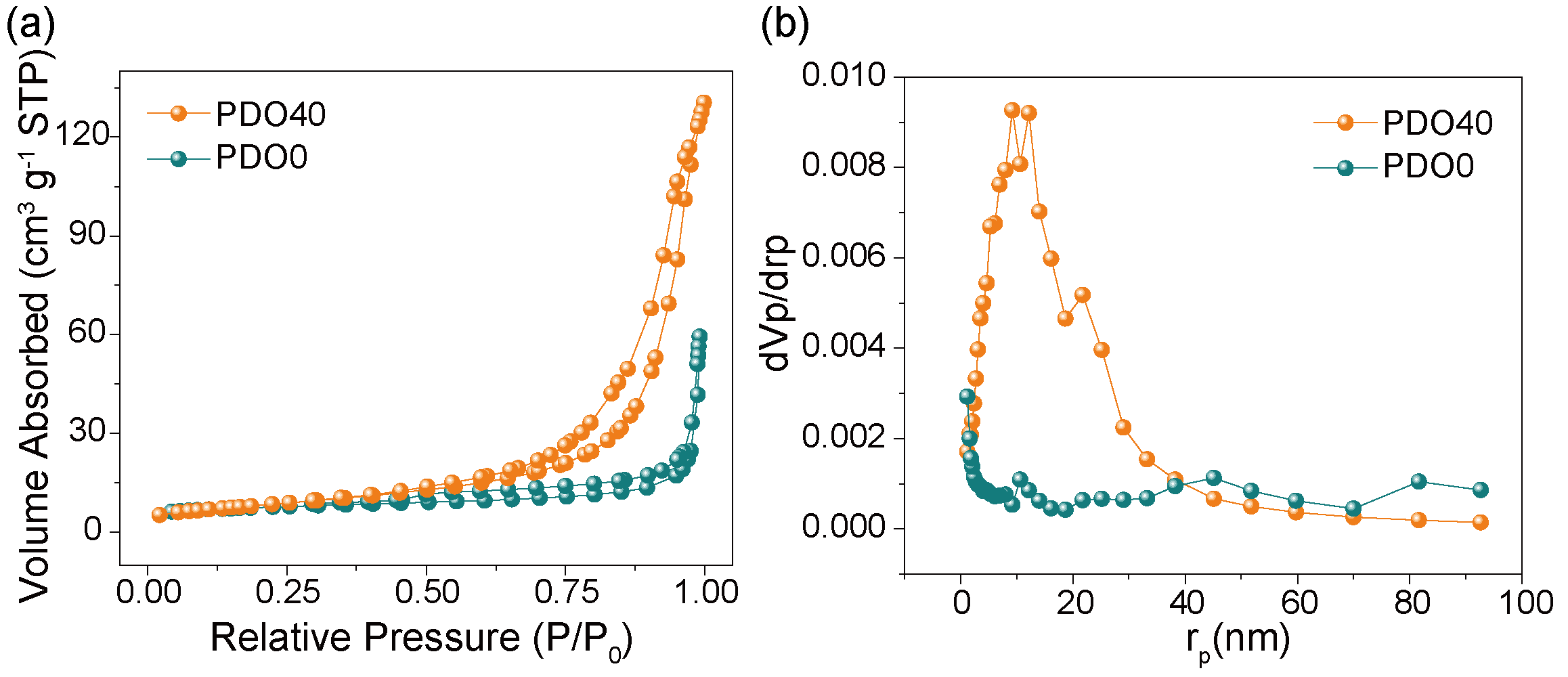


1. (a) Nitrogen adsorption-desorption isotherms. (b) The corresponding pore size distribution curves.


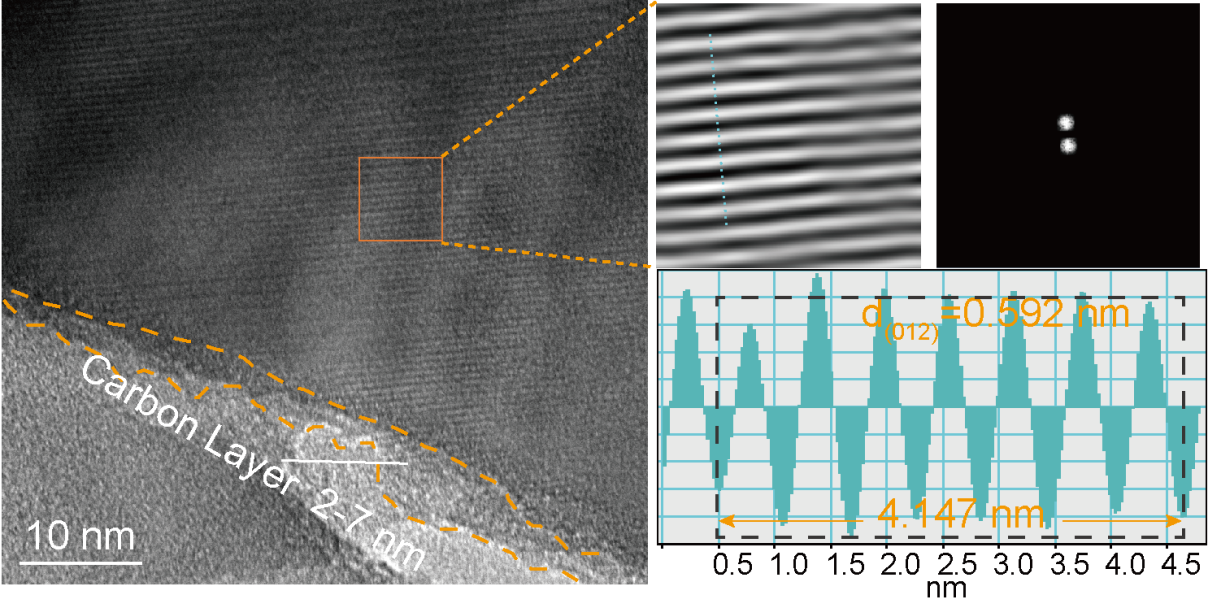


1. HR-TEM image with the corresponding FFT pattern in the top-right and an intensity line profile in the bottom-right.


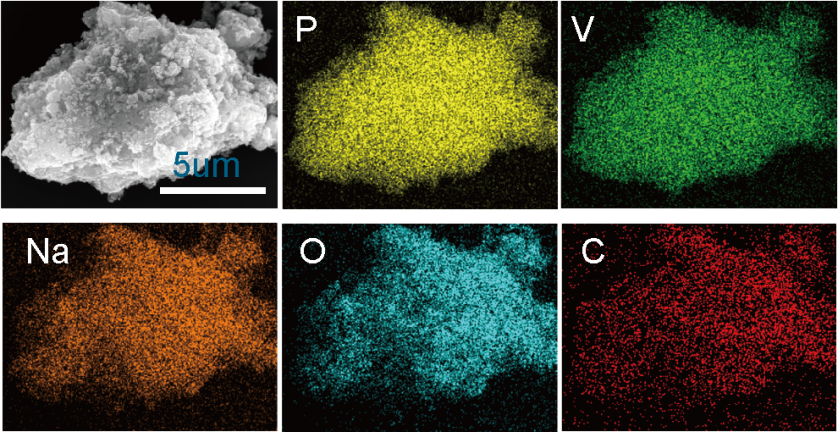


1. EDS mapping images of PDO40.


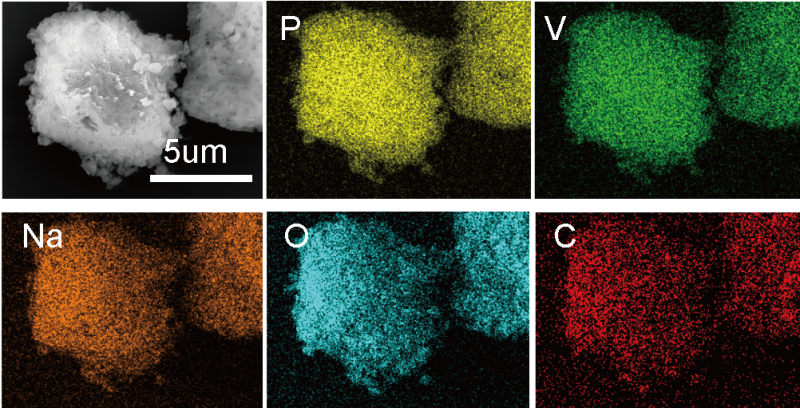


1. EDS mapping images of PDO0.


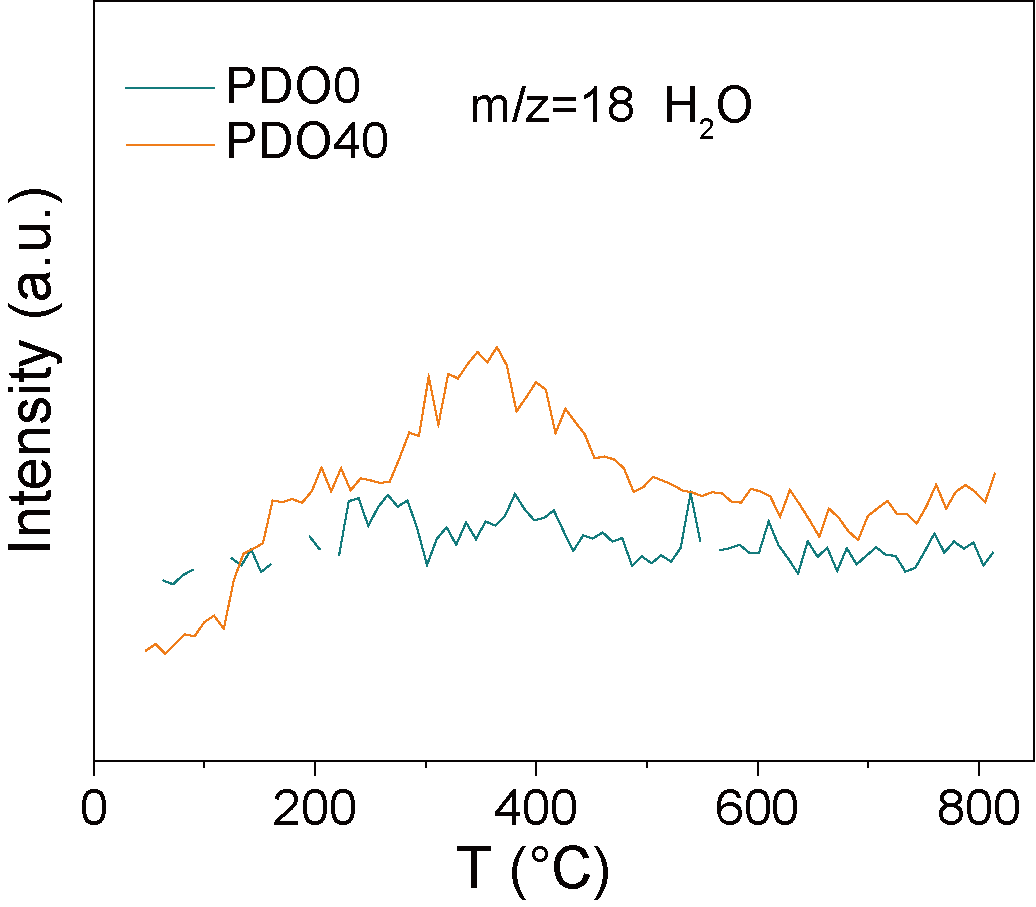


1. The evolution profiles of pyrolytic H_2_O release as a function of temperature from TG-MS.


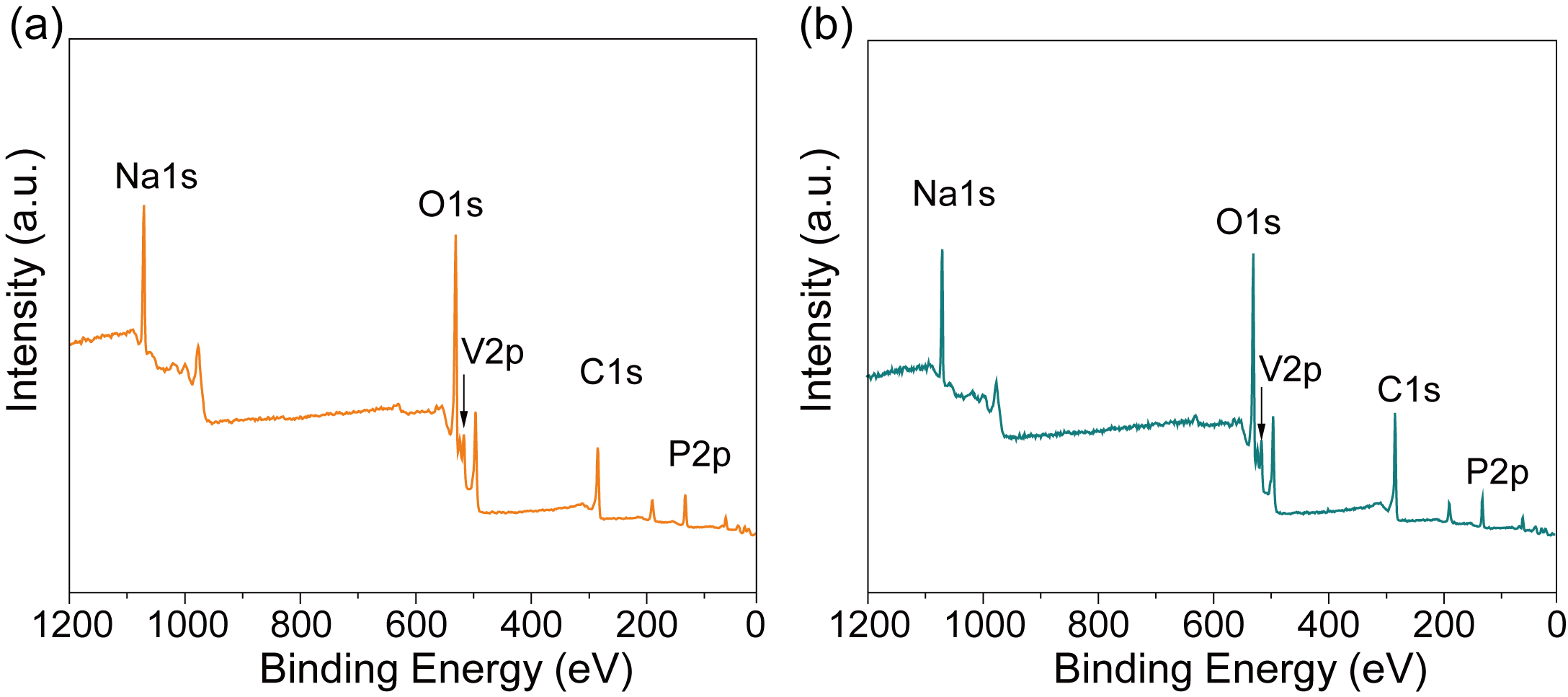


1. The full-scale spectra XPS of (a) PDO40 and (b) PDO0.


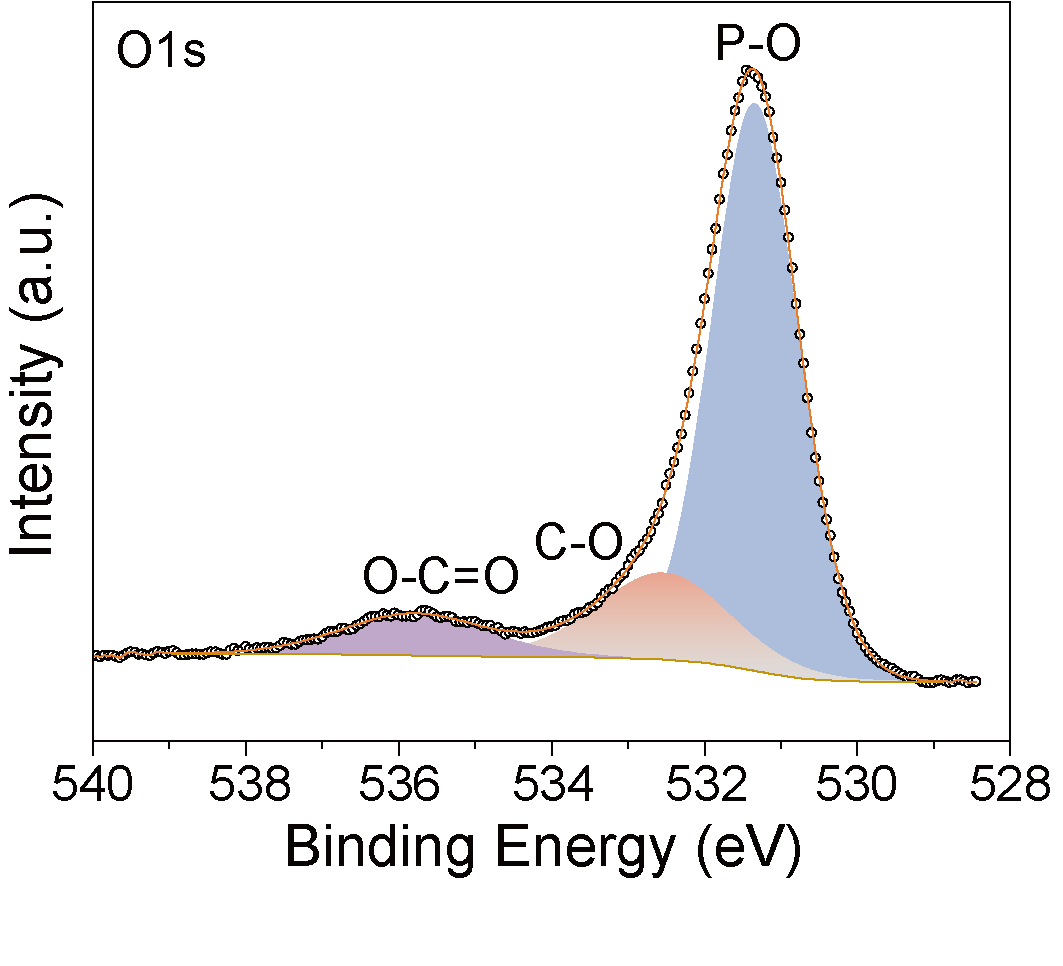


1. XPS spectra of O 1s in PDO0.


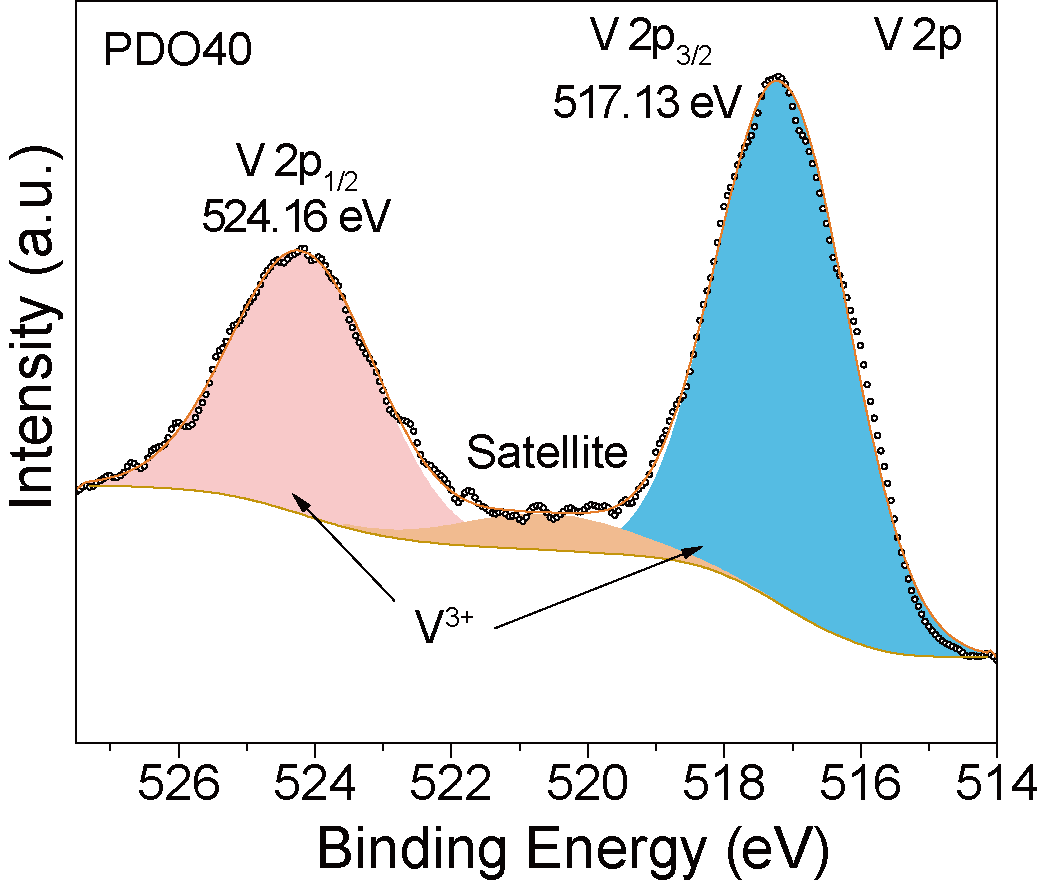


1. XPS spectra of V 2p in PDO40.


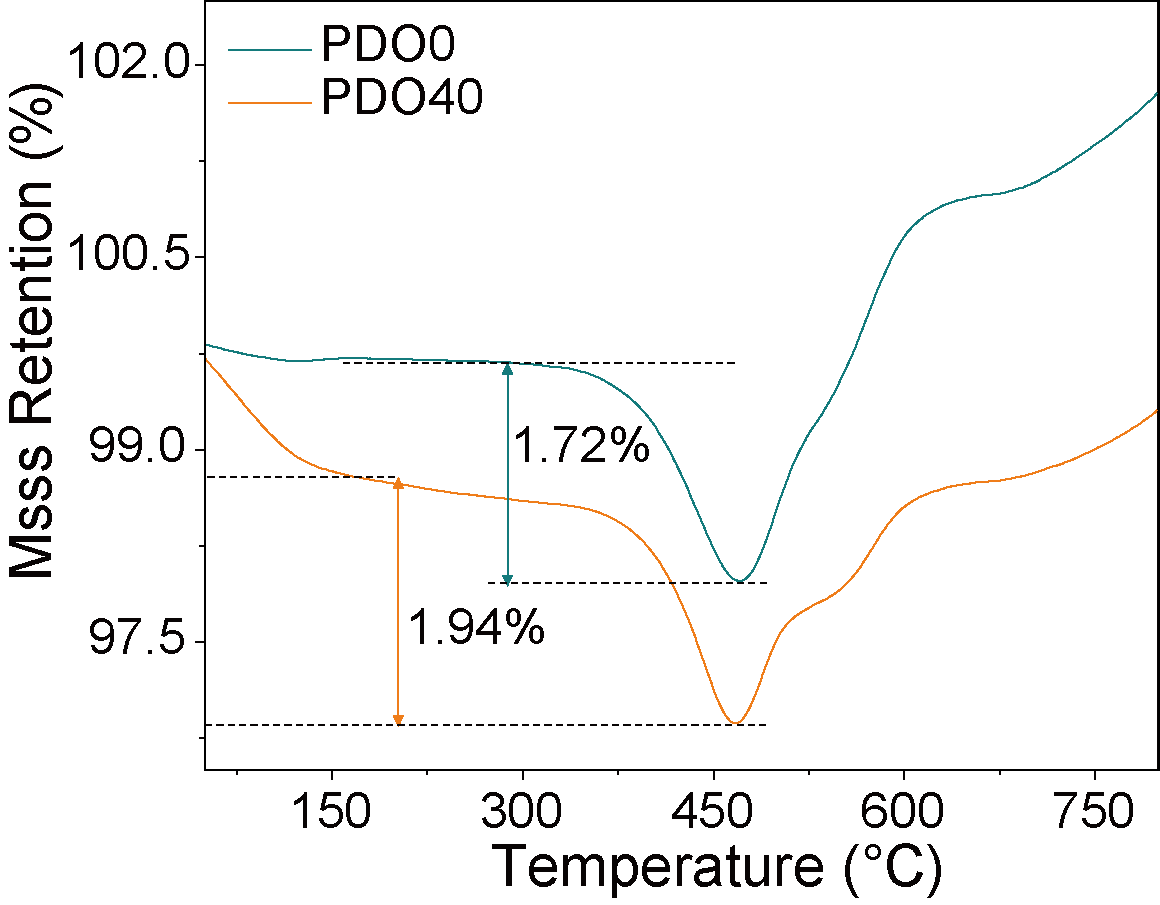


1. TG curves.


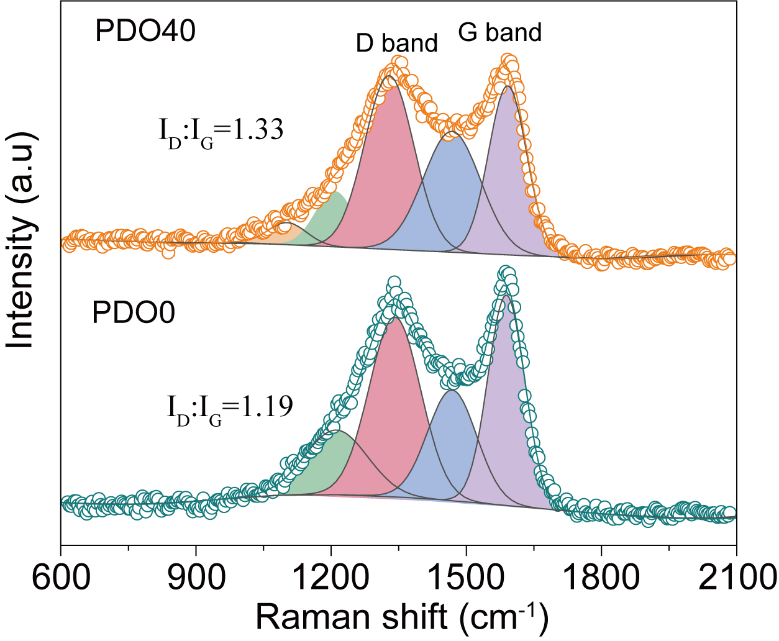


1. Raman spectrogram of PDO0 and PDO40.


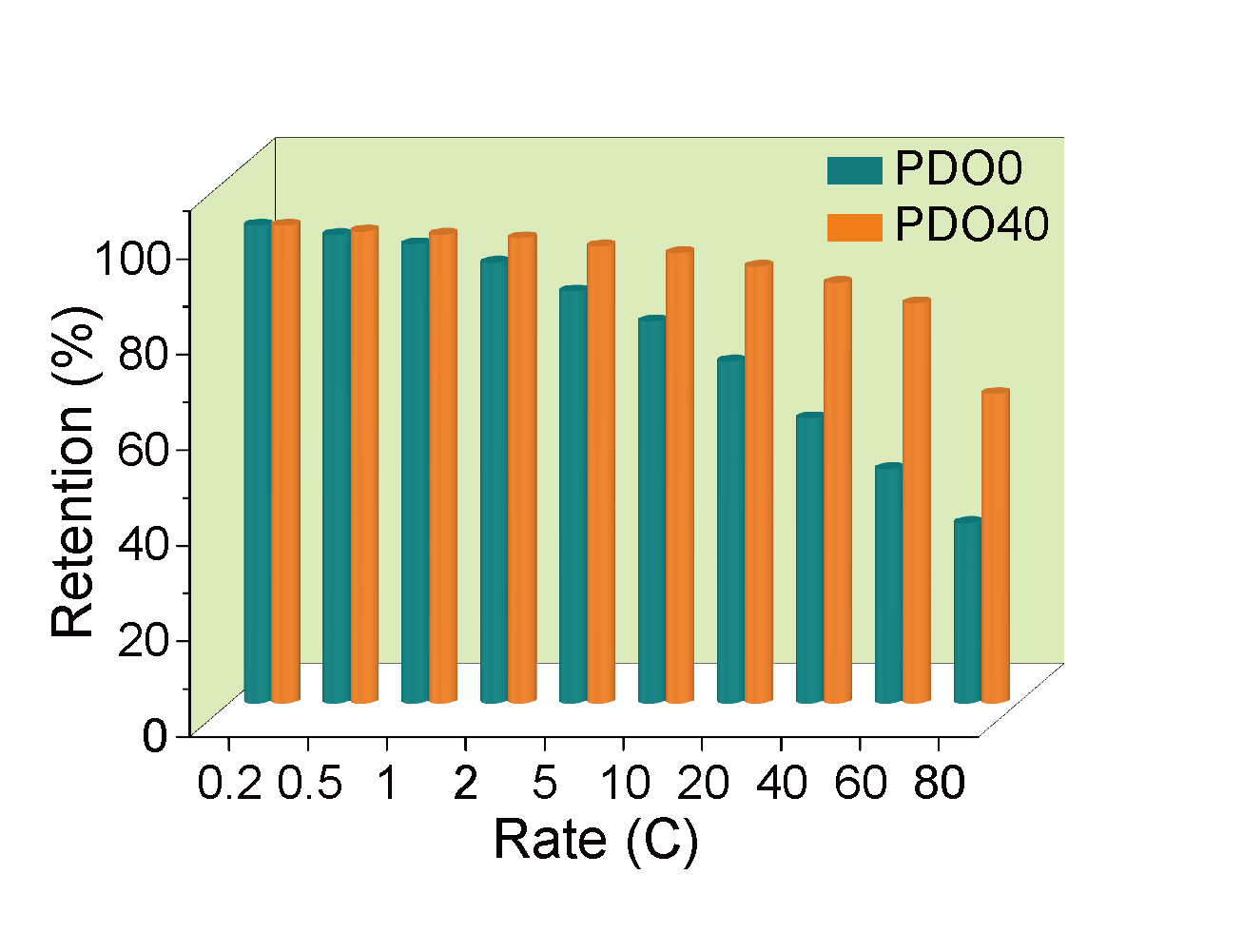


1. Capacity retention at different rates.


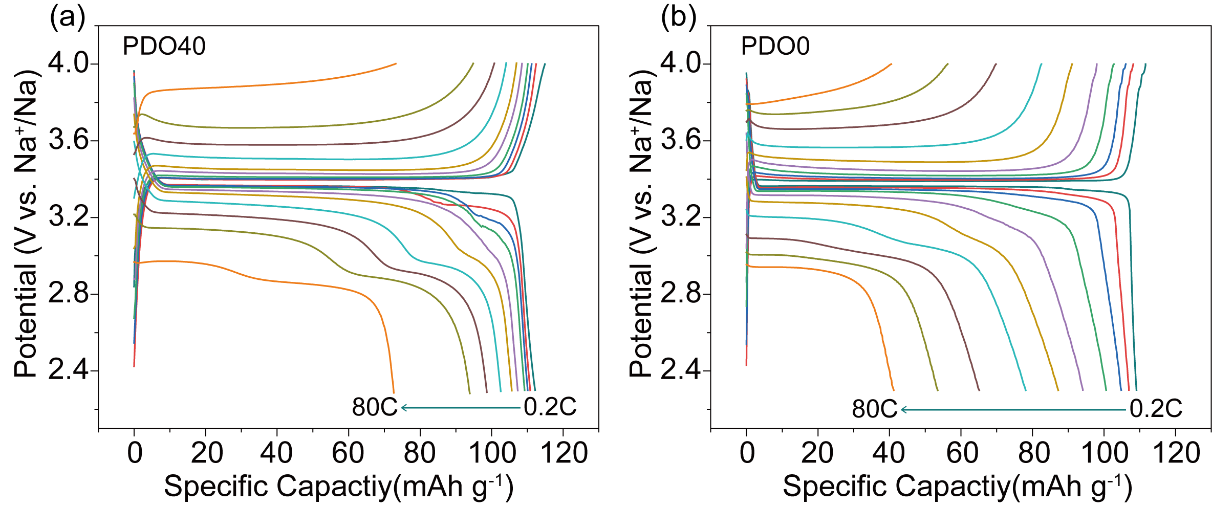


1. GCD curves at different rate. (a) PDO40. (b) PDO0.


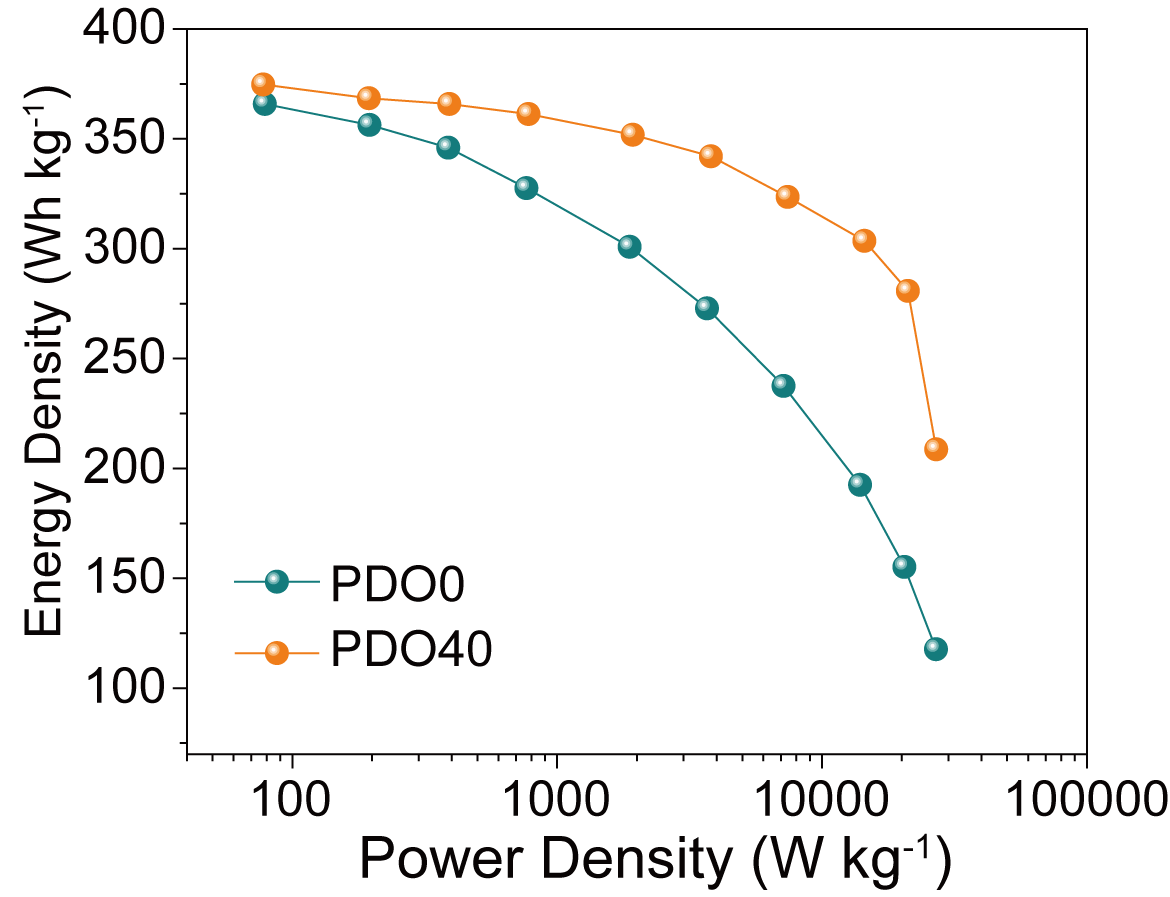


1. Ragone plot of cathodes from 0.2 C to 80 C


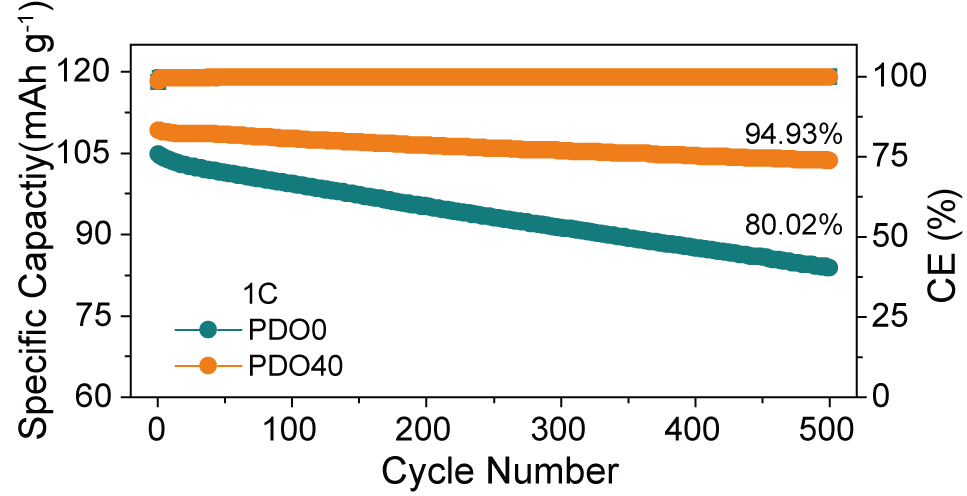


1. Cycling performance at 1C.


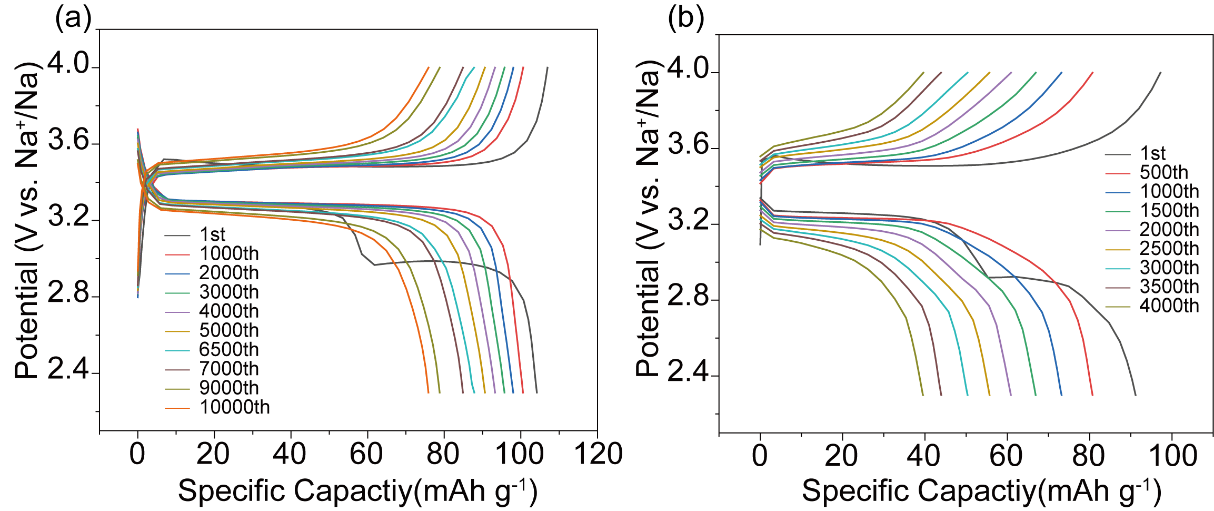


1. GCD curves of 10C at different cycle numbers.


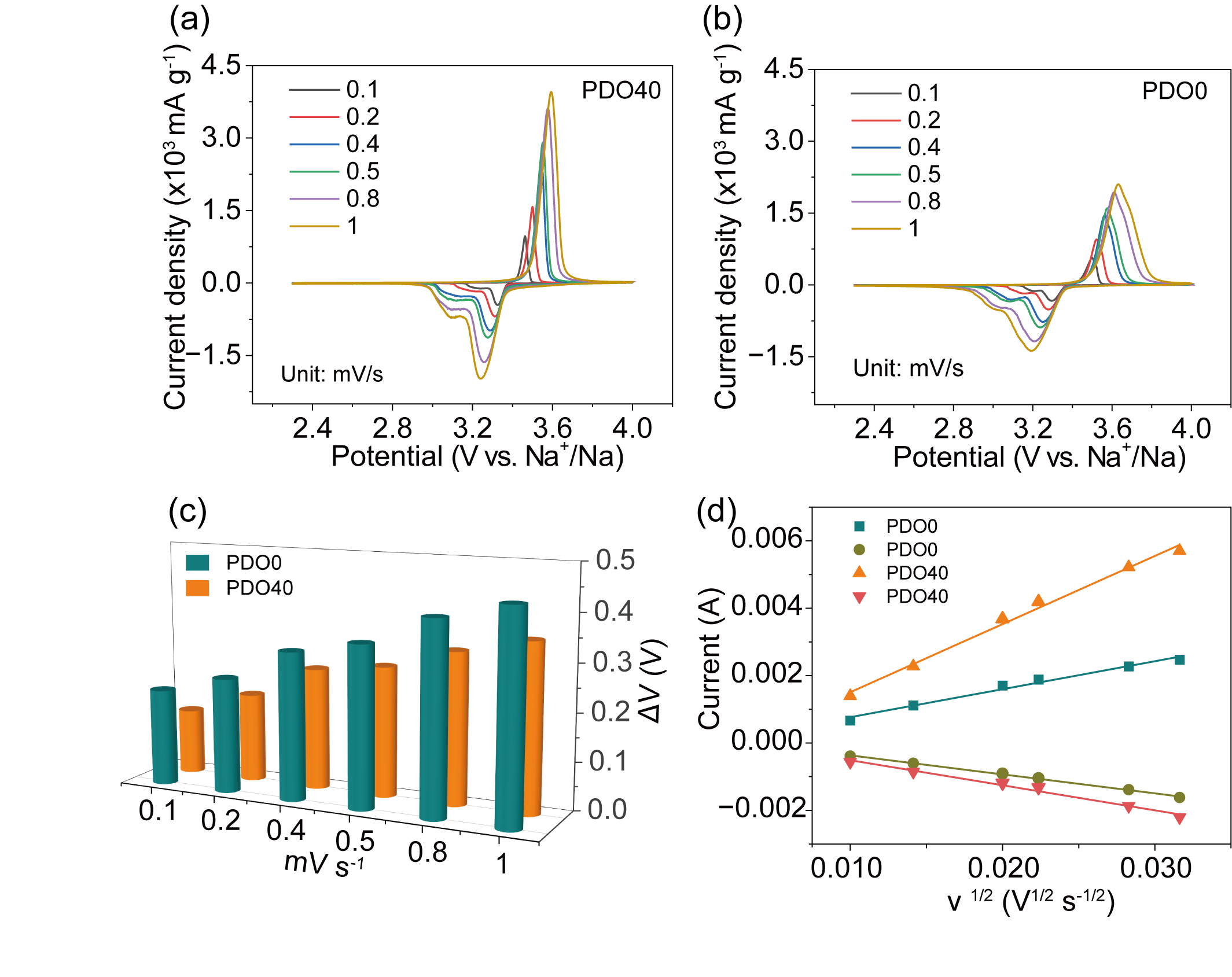


1. CV curves at different scan rates (a) PDO40. (b) PDO0. (c) oxidation-reduction potential difference at different scan rates. (d) Corresponding linear fit curves of Ip versus v^1/2^.


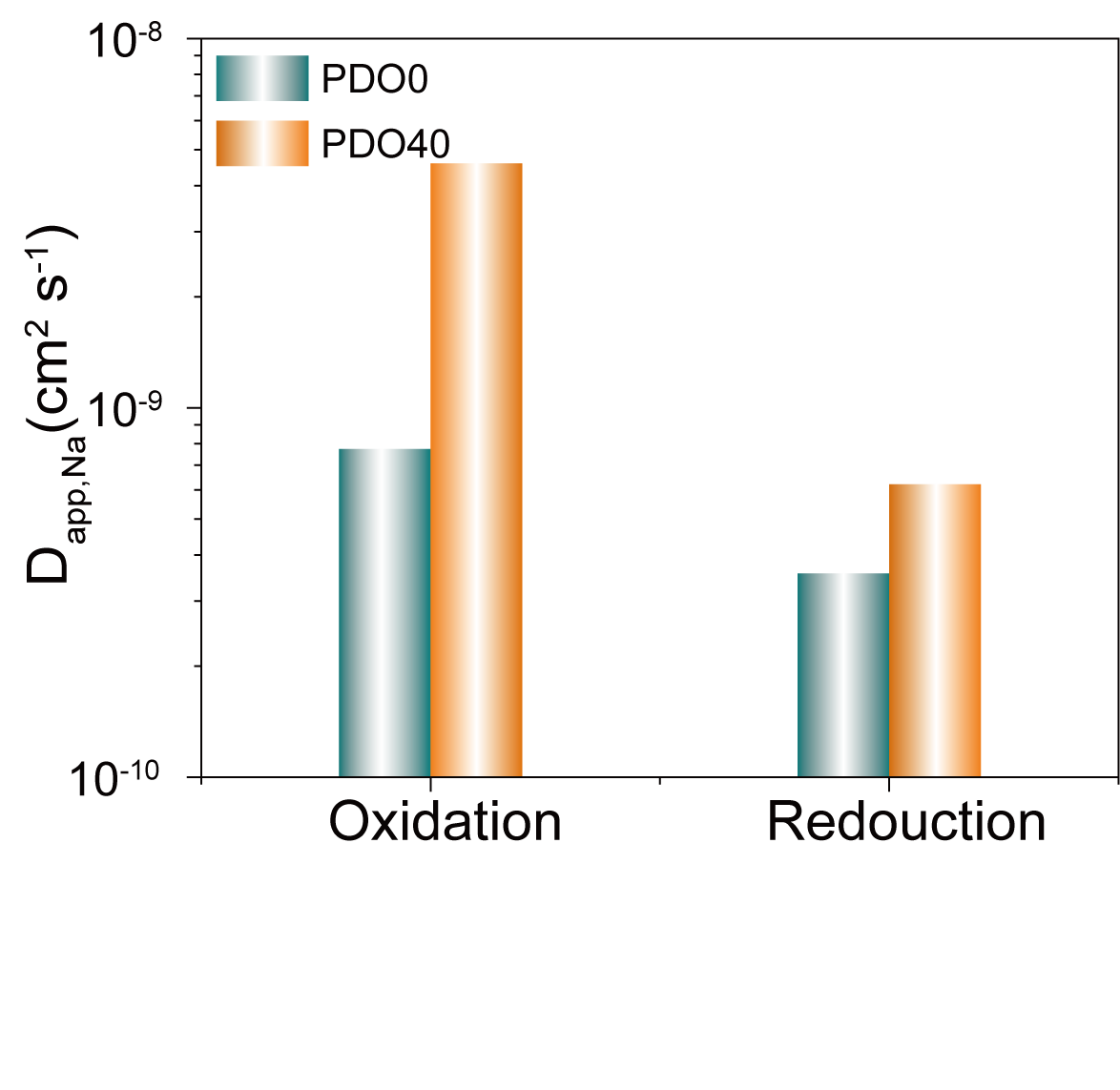


1. *D*_app,Na_ values of redox peak calculated from CV curves.


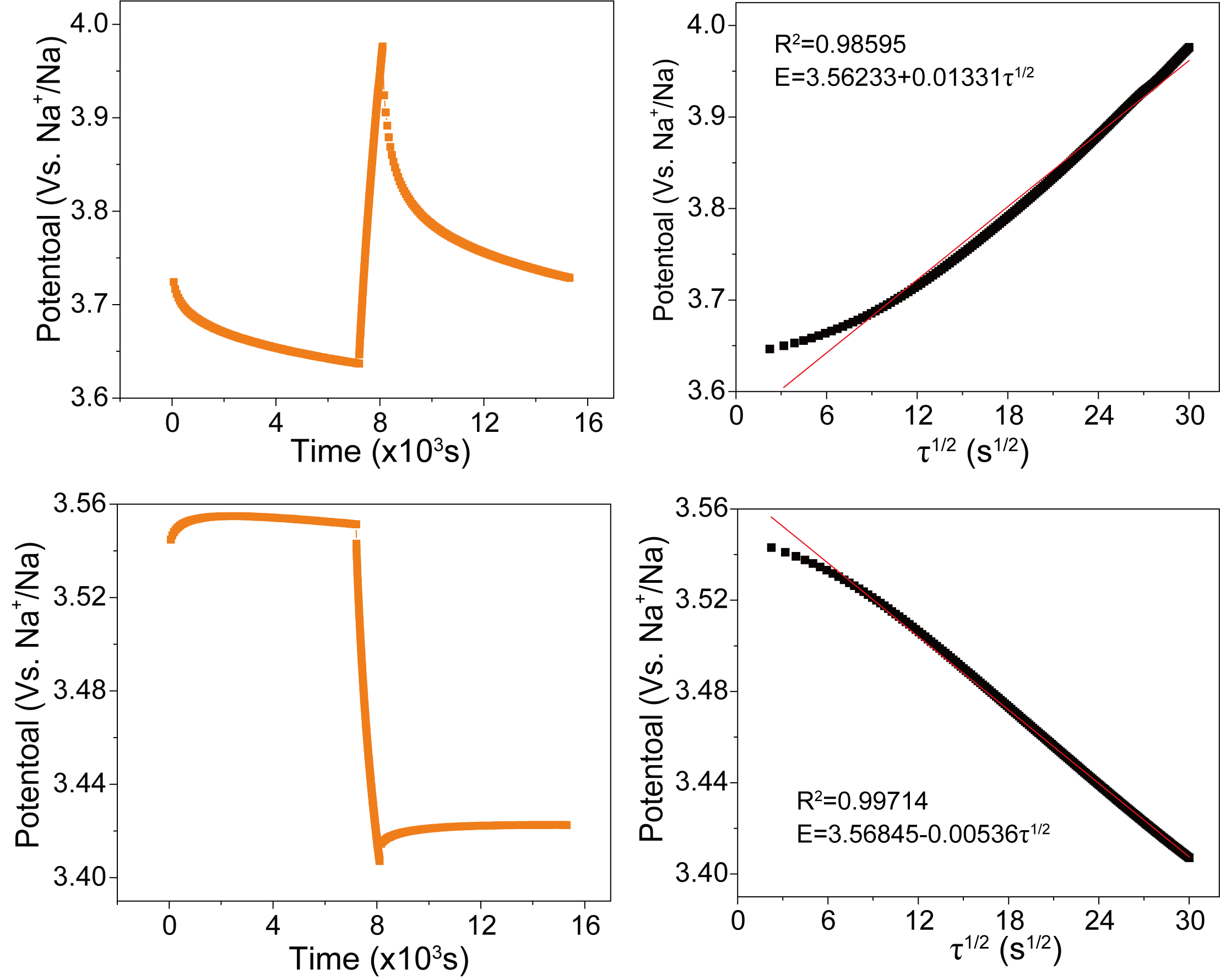


1. The relationship between τ and E during a single GITT process and the linear fitting of E and τ^1/2^ for the PDO40 half-cell. Charging process. (a) and (b). Discharging process. (c) and (d).


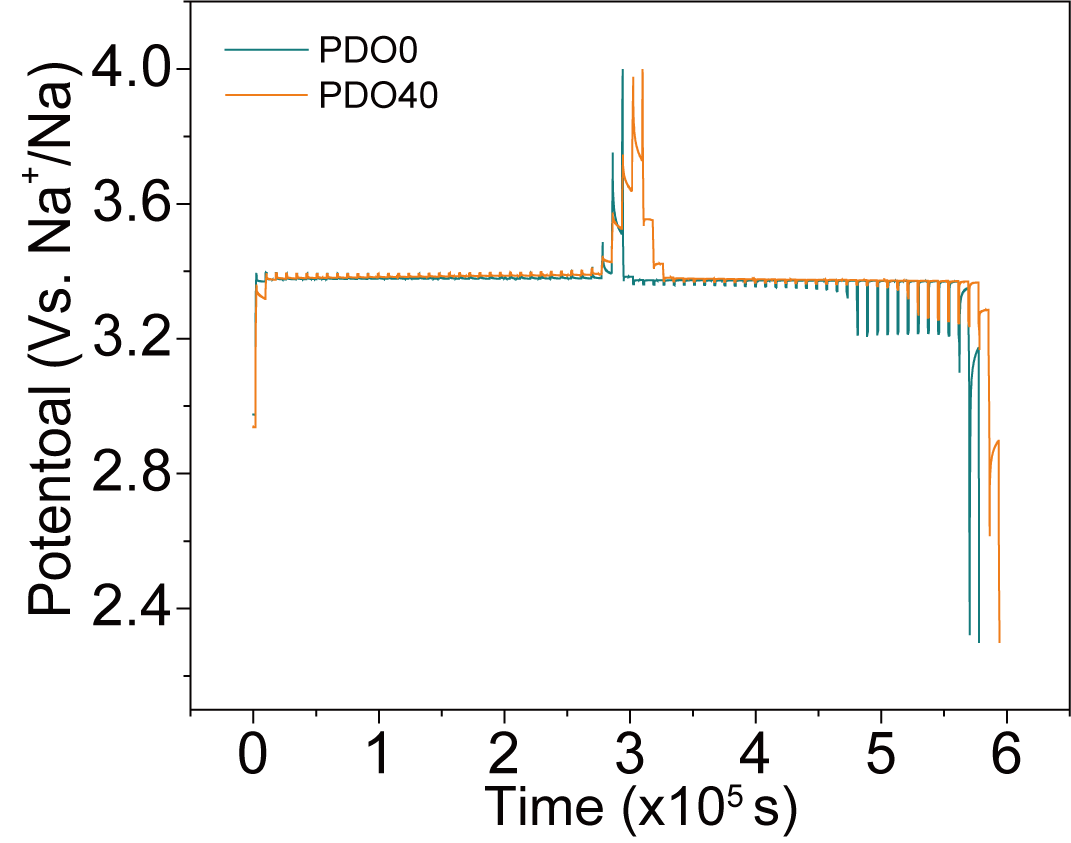


1. GITT curves of PDO0 and PDO40.


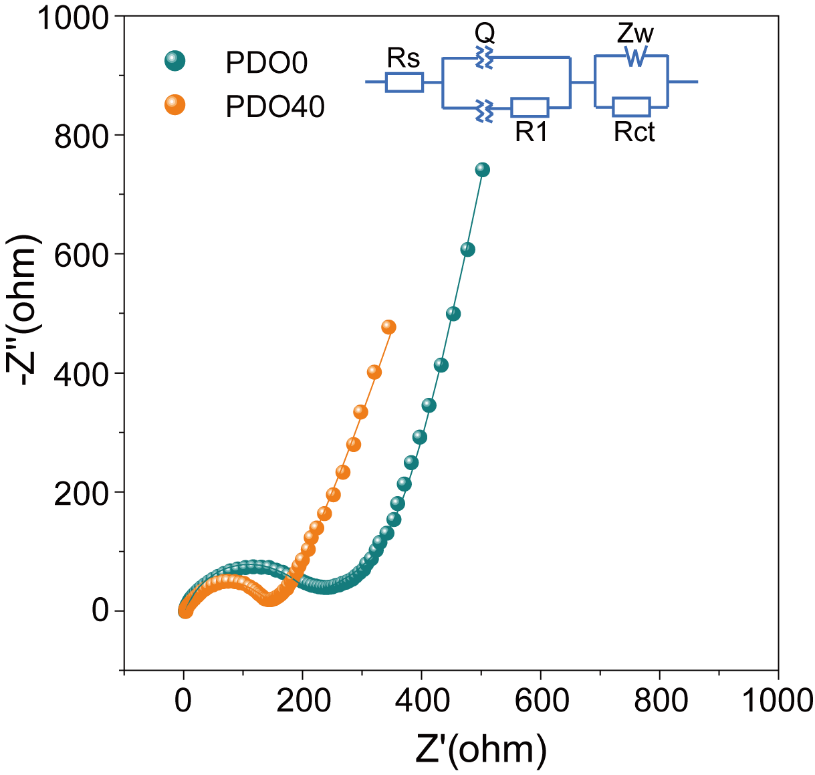
{Bai, 2024 #32}

1. The Nyquist plots of PDO0 and PDO40 electrodes in their pristine state, with an inset showing the equivalent circuit model used for fitting the EIS data.


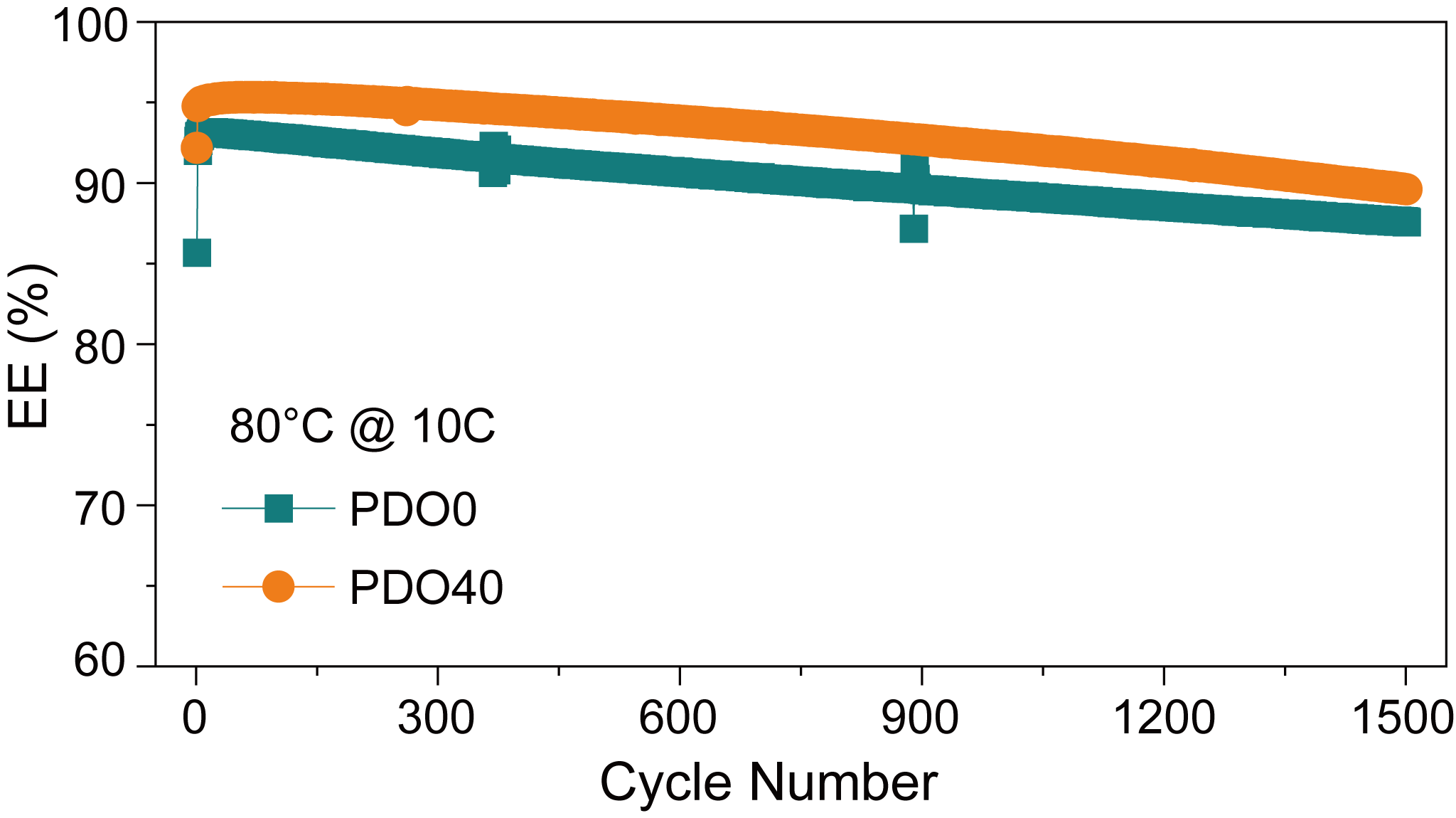


1. The energy efficiency of cycling at 10 C under 80 °C.


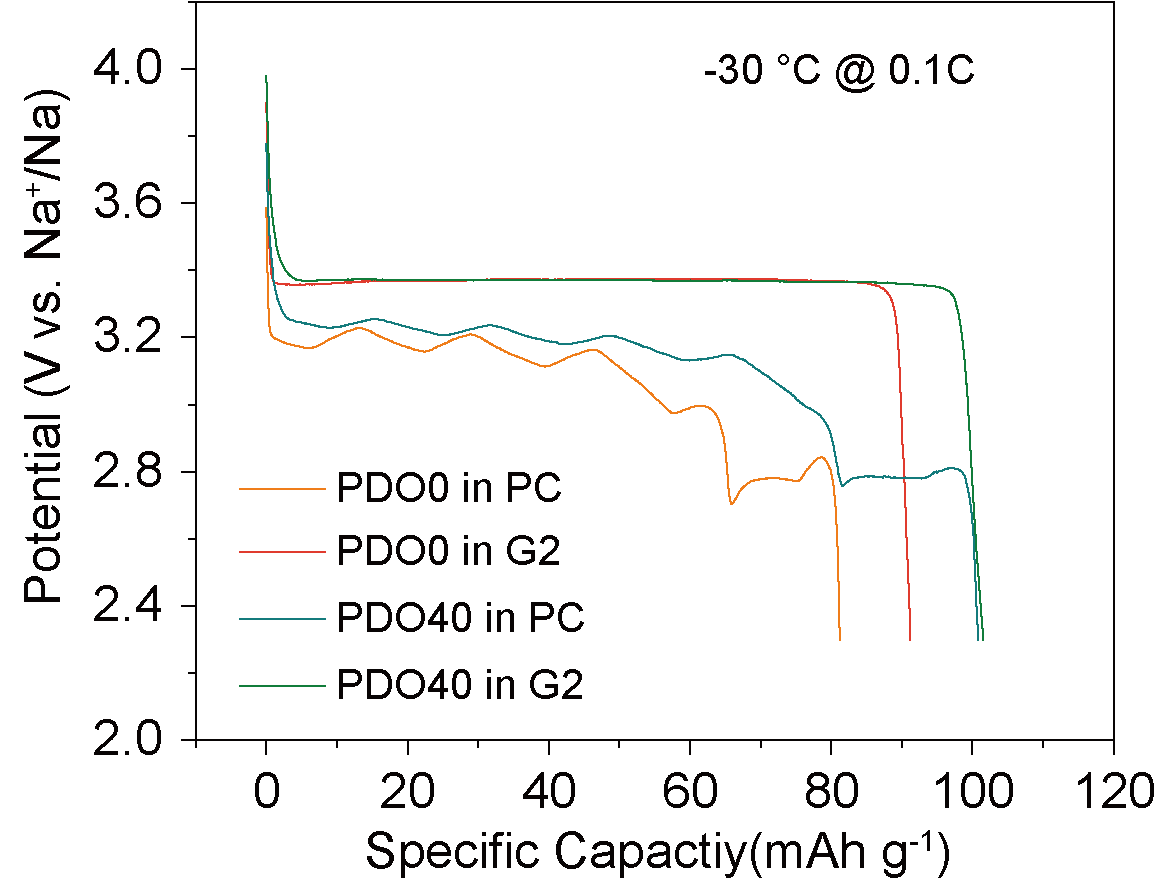


1. Discharge curves at 0.1 C with different electrolytes at -30 °C.


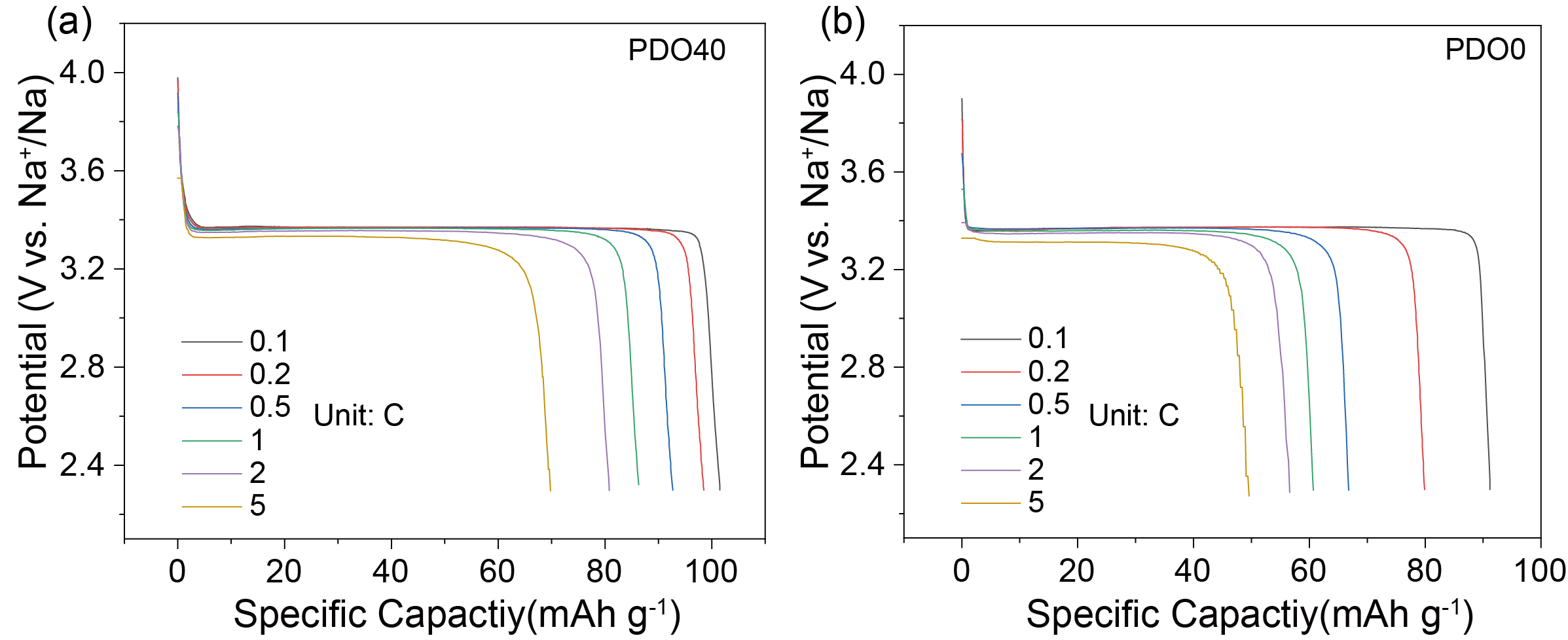


1. Discharge curves at different rates at -30 °C.(a) PDO40. (b) PDO0.


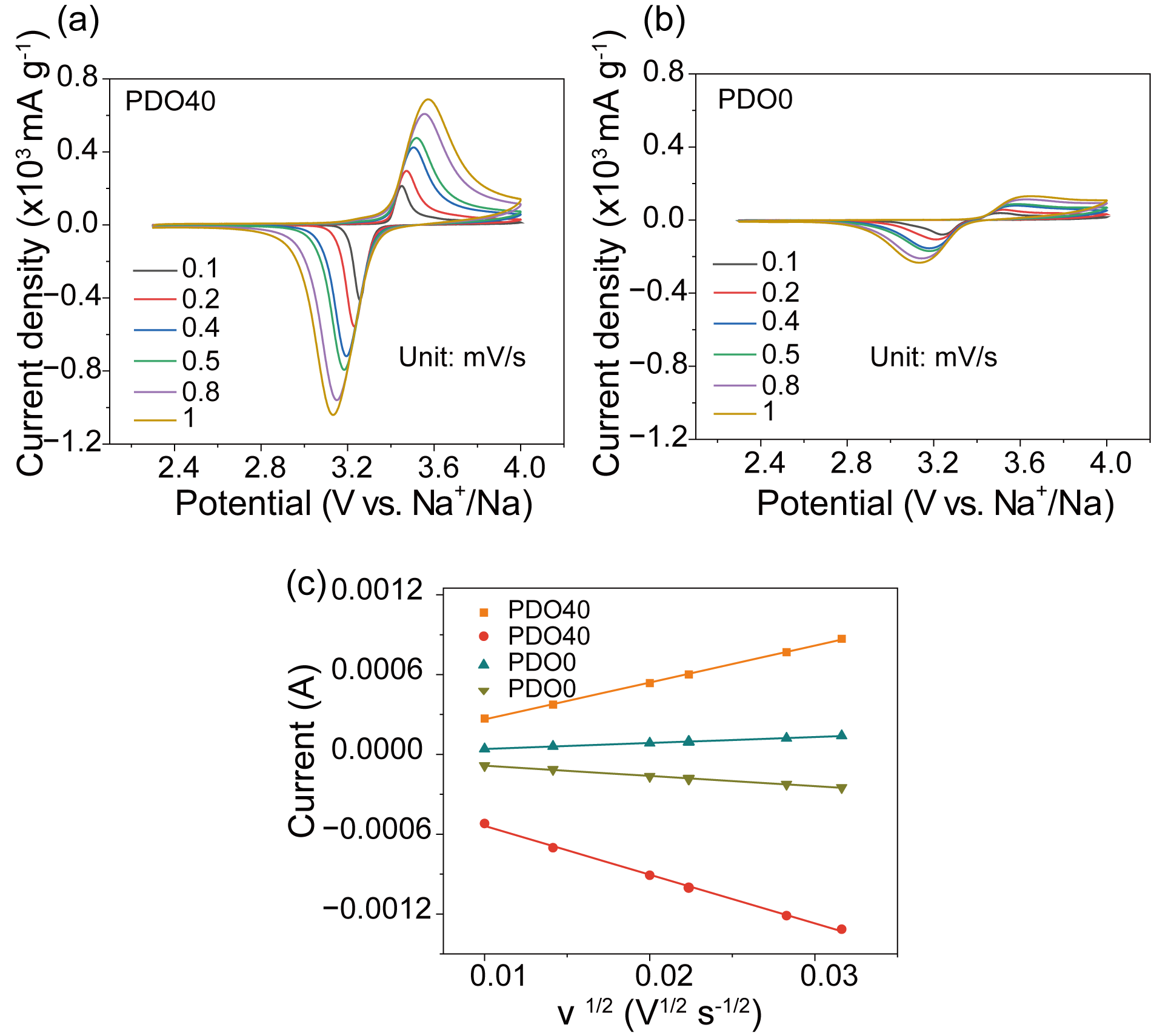


1. CV curves at different scan rates at -60 °C (a) PDO40. (b) PDO0. (c) Corresponding linear fit curves of Ip versus v^1/2^.


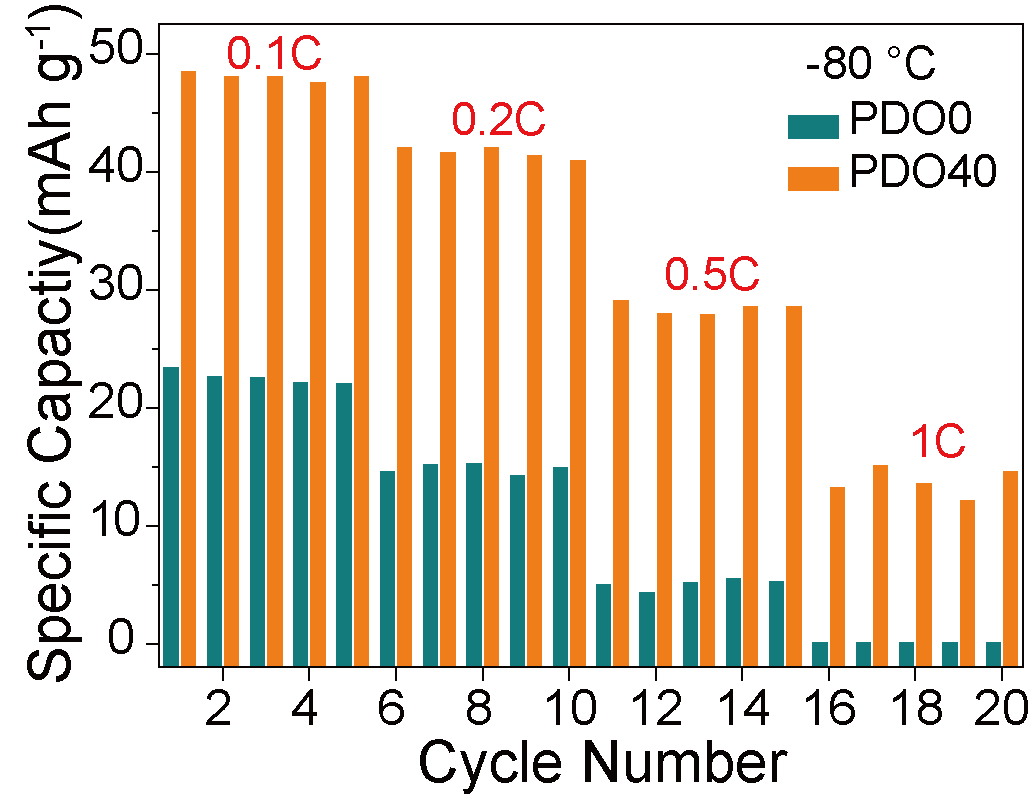


1. Rate performance at -80 °C.


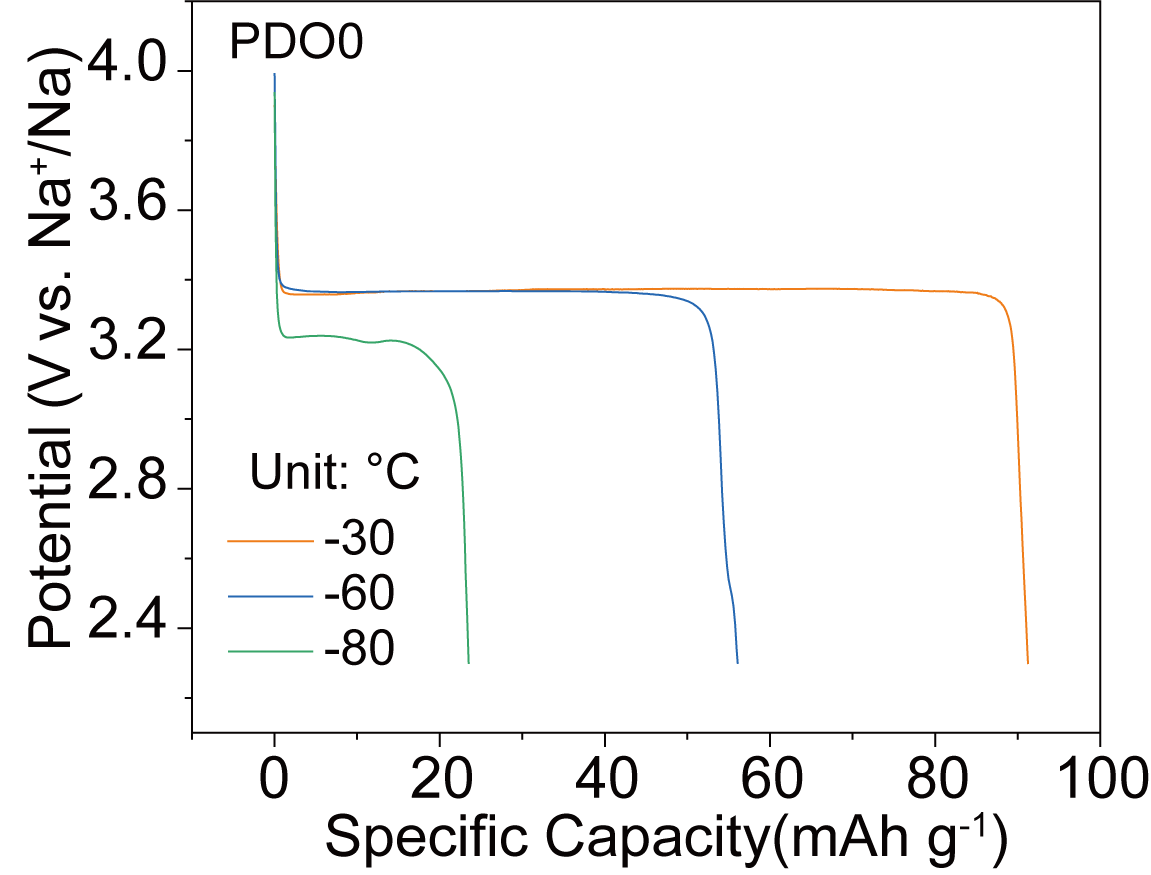


1. Discharge curves at 0.1C at different low-temperatures of PDO0.


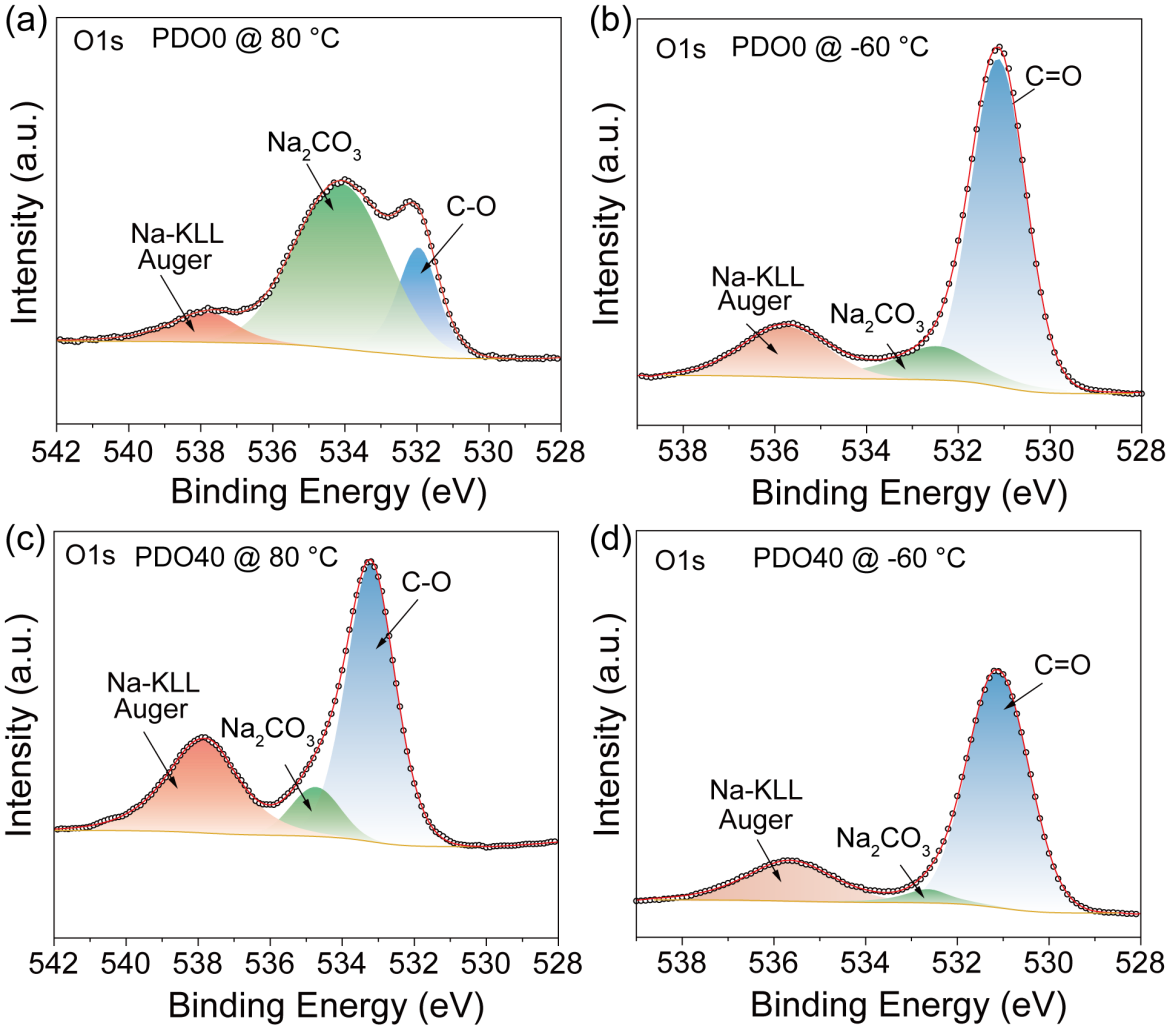


1. XPS spectra of F 1s of PDO0 and PDO40 after cycling at different temperatures. (a)PDO0 at 80 °C. (b) PDO40 at 80 °C. (c)PDO0 at -60 °C. (d) PDO40 at -60 °C.


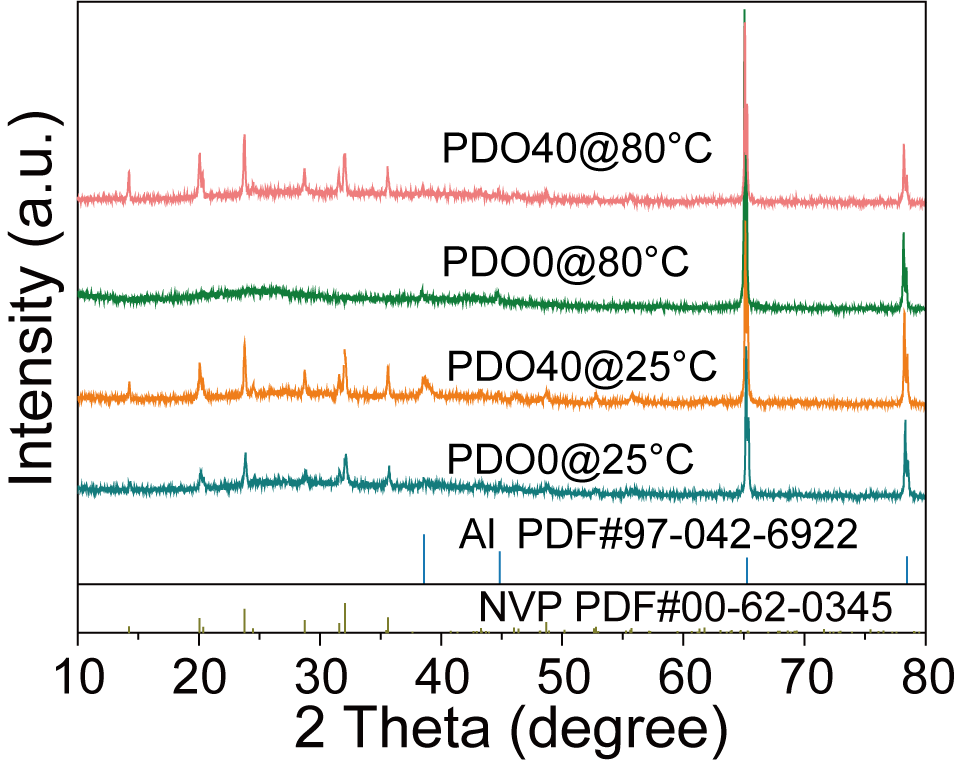


1. XRD of PDO0 and PDO40 after cycling at different temperatures.


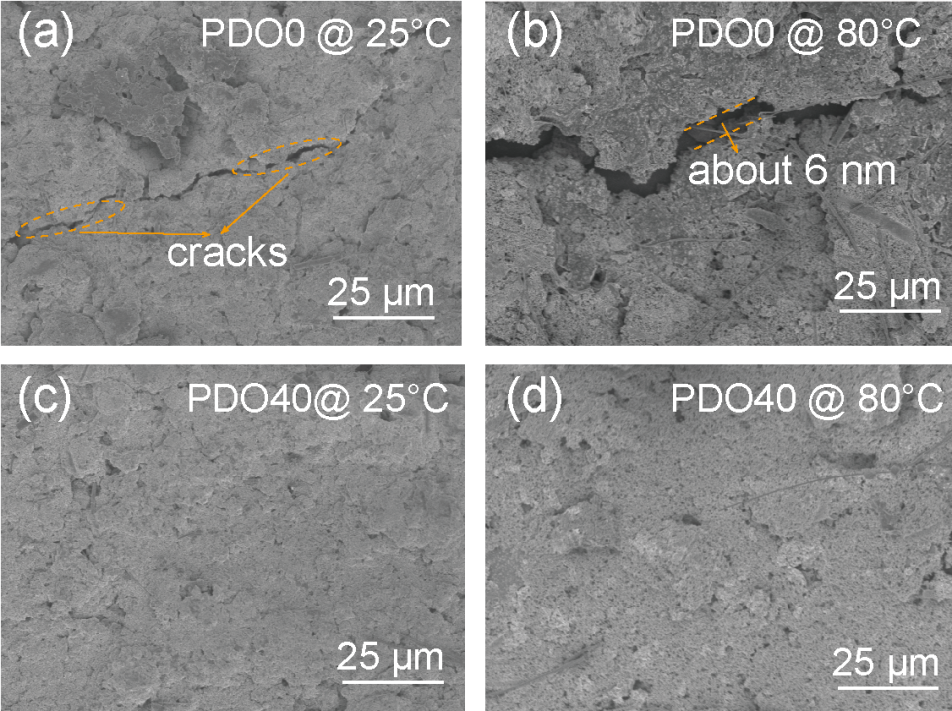


1. SEM of PDO0 and PDO40 after cycling at different temperatures.


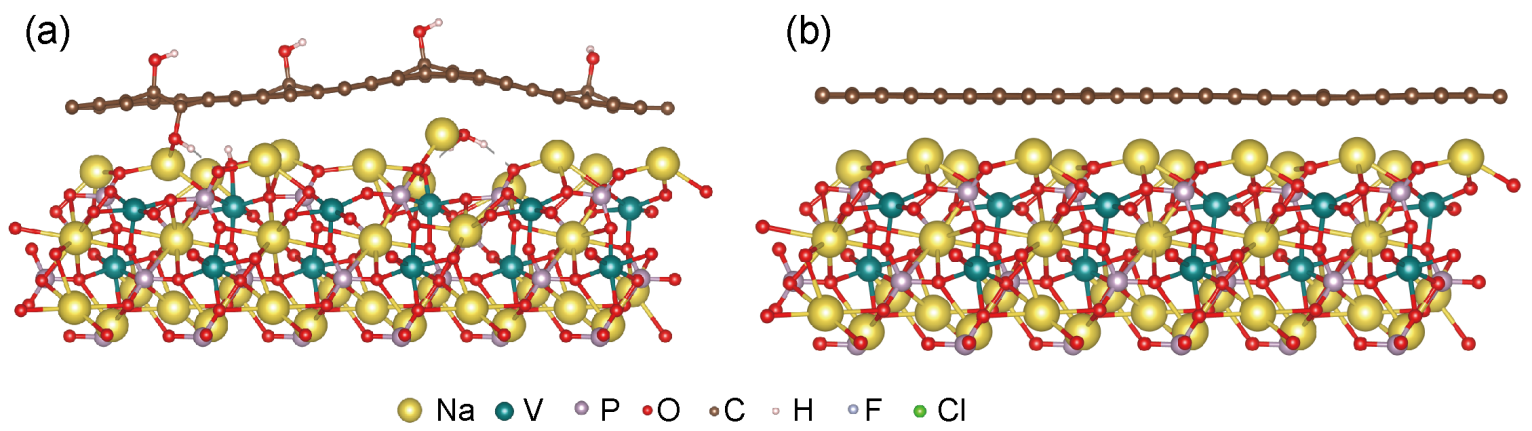


1. Structural modeling of PDO40 and PDO0.


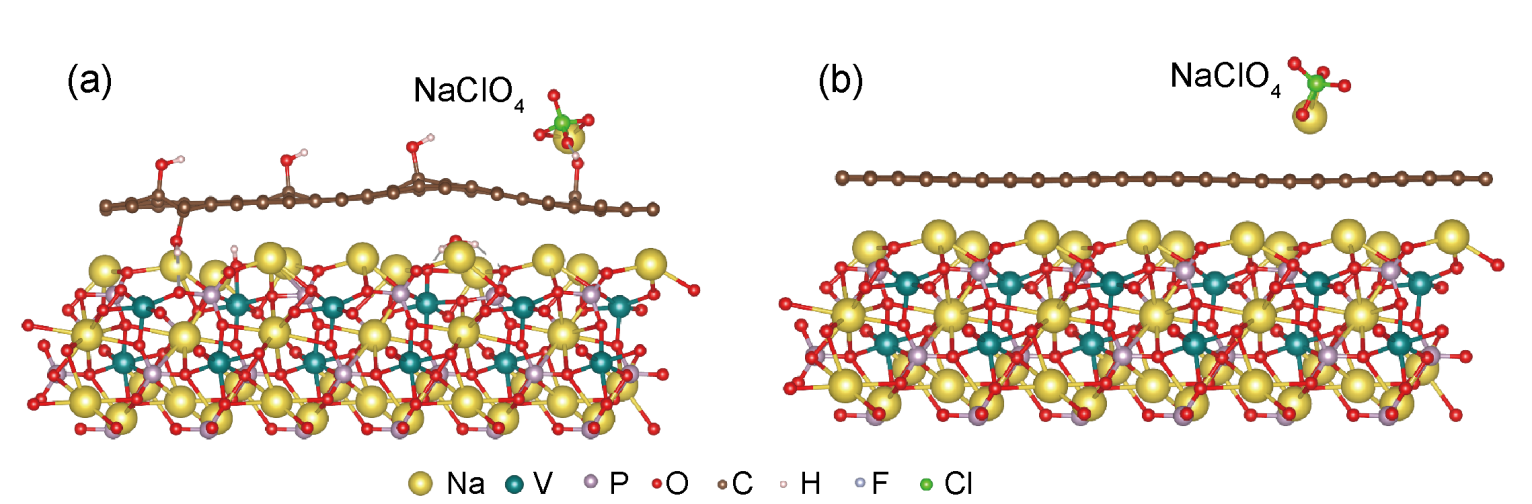


1. Structural modeling of after adsorption with NaClO_4_.


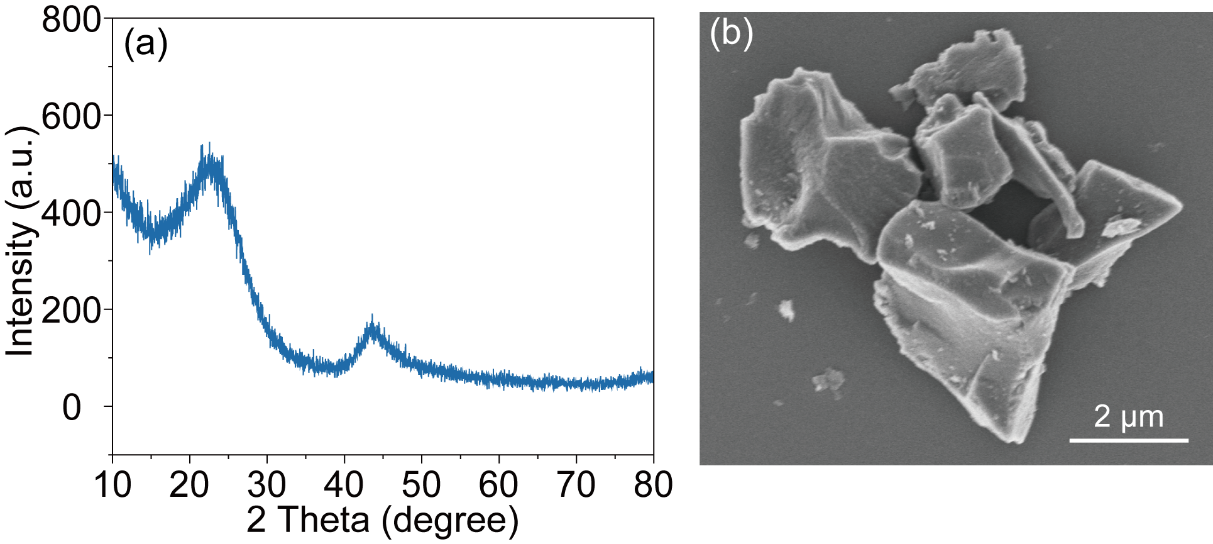


1. The SEM image and XRD patterns of HC.


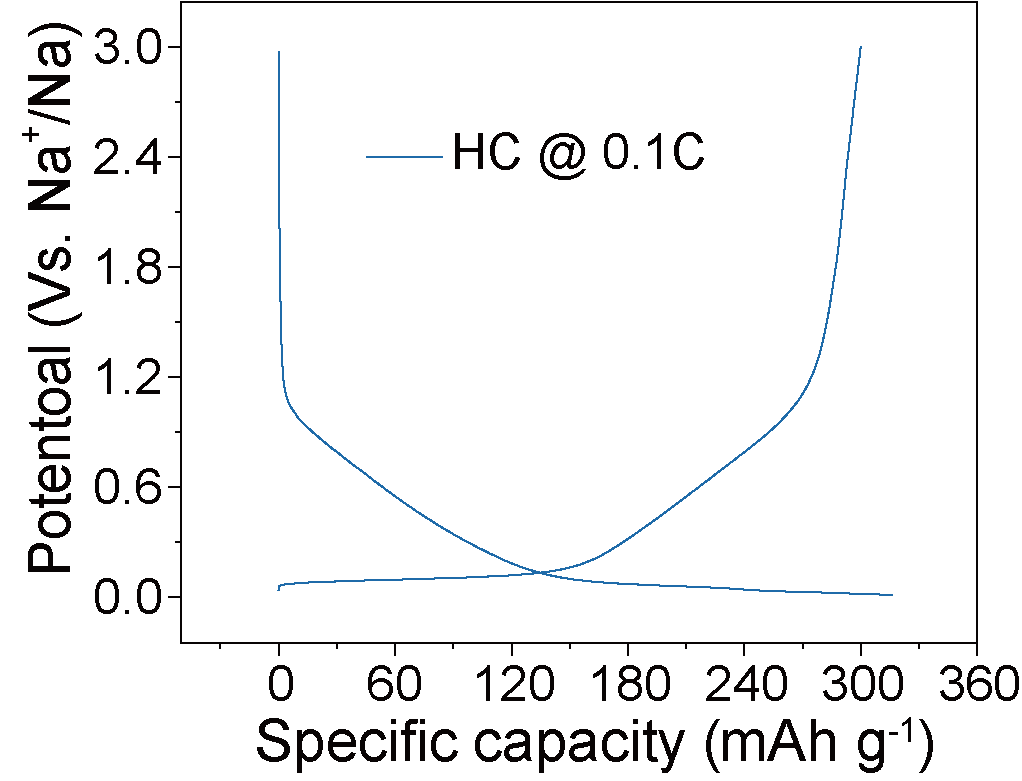


1. GCD curves of HC in 0.01-3 V.


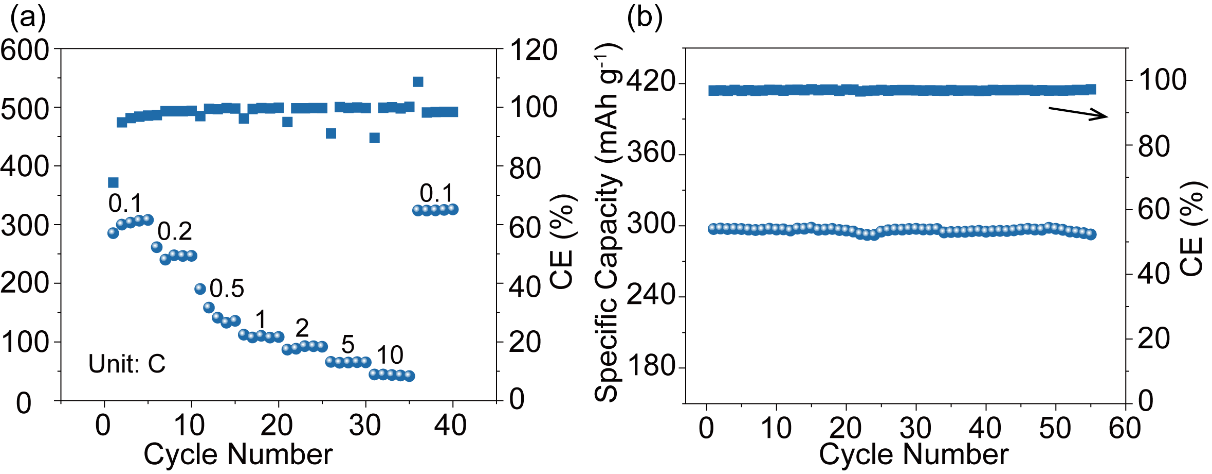


1. The electrochemical performance of HC. (a) Rate performance. (b) The cycling performance @ 0.1C

**Table S1.** Lattice parameters and evaluation parameters obtained from Rietveld refinement of XRD.

| sample | Lattice parameters | | | | Evaluation parameters | | |
| --- | --- | --- | --- | --- | --- | --- | --- |
|  | a(Å) | b(Å) | c(Å) | V(Å^3^) | Rwp | Rp | Rexp |
| PDO0 | 8.73230 | 8.73230 | 21.79180 | 1439.06696 | 7.46% | 5.06% | 1.93% |
| PDO40 | 8.73160 | 8.73160 | 21.82120 | 1440.77743 | 5.10% | 3.62% | 1.76% |

**Table S2.** Refined structural parameters of the PDO0 cathode from XRD Rietveld refinements.

| Atom | Type | Wyckoff | X | Y | Z | Occupancy |
| --- | --- | --- | --- | --- | --- | --- |
| Na | Na1 | 6b | 0.33333 | 0.66667 | 0.16667 | 0.805 |
| Na | Na2 | 18e | 0.66667 | 0.96210 | 0.08333 | 0.731 |
| V | V1 | 12c | 0.33333 | 0.66667 | 0.01896 | 1.000 |
| P | P1 | 18e | -0.04430 | 0.33333 | 0.08333 | 1.000 |
| O | O1 | 36f | 0.13940 | 0.49980 | 0.08030 | 1.000 |
| O | O2 | 36f | 0.54820 | 0.84570 | -0.02640 | 1.000 |

**Table S3.** Refined structural parameters of the PDO40 cathode from XRD Rietveld refinements.

| Atom | Type | Wyckoff | X | Y | Z | Occupancy |
| --- | --- | --- | --- | --- | --- | --- |
| Na | Na1 | 6b | 0.33333 | 0.66667 | 0.16667 | 0.805 |
| Na | Na2 | 18e | 0.66667 | 0.96210 | 0.08333 | 0.731 |
| V | V1 | 12c | 0.33333 | 0.66667 | 0.01896 | 1.000 |
| P | P1 | 18e | -0.04430 | 0.33333 | 0.08333 | 1.000 |
| O | O1 | 36f | 0.13940 | 0.49980 | 0.08030 | 1.000 |
| O | O2 | 36f | 0.54820 | 0.84570 | -0.02640 | 1.000 |

Reference

[1] G. Kresse; D. Joubert, *Phys. Rev. B* **1999**, *59*, 1758-1177.

[2] John P. Perdew; K. Burke; M. Ernzerhof, *Physical Review Letters* **1996**, *77*, 3865-3868.
